# Genetic mutations governing ferroptosis sensitivity and resistance: a precision approach to cancer therapy

**DOI:** 10.1038/s41419-026-08796-w

**Published:** 2026-05-07

**Authors:** Peyman Tabnak, Mohammad Ebrahimnezhad, Zanyar HajiEsmailPoor

**Affiliations:** 1 Immunology Research Center, Tabriz University of Medical Sciences, Tabriz, Iran; 2 Department of Clinical Biochemistry and Laboratory Medicine, Faculty of Medicine, Tabriz University of Medical Sciences, Tabriz, Iran

**Keywords:** Cancer therapeutic resistance, Cancer genomics, Targeted therapies

## Abstract

Ferroptosis, an iron-dependent programmed cell death pathway driven by lipid peroxidation, offers a transformative approach to cancer therapy by exploiting unique cellular vulnerabilities. This comprehensive review elucidates the intricate molecular mechanisms of ferroptosis and their modulation by genetic mutations across diverse malignancies, including lung, hematological, liver, colorectal, breast, glioma, renal, pancreatic, thyroid, prostate, cervical, gastric, and melanoma. We delineate the critical functions of ferroptosis regulators, such as GPX4, system Xc⁻, and iron metabolism proteins, in orchestrating the delicate balance between oxidative damage and antioxidant protection. The study further examines how oncogenic mutations in genes like EGFR, KRAS, TP53, KEAP1, and IDH1 reshape ferroptosis susceptibility or resistance through alterations in metabolic pathways, redox homeostasis, and tumor microenvironment interactions. By highlighting mutation-specific sensitivities, this work underscores the potential of ferroptosis-targeted strategies to surmount therapeutic resistance, synergize with conventional treatments like chemotherapy and immunotherapy, and drive precision oncology forward, paving the way for enhanced clinical outcomes across a broad spectrum of cancers.

## Introduction

Ferroptosis is a programmed form of cell death that involves iron-catalyzed lipid peroxidation, primarily in cell membranes. Unlike most forms of cell death, implementation depends on the accumulation of oxidized polyunsaturated fatty acids, which ruptures membrane integrity and lead to cell lysis [1]. This is an important process since it contributes to tissue homeostasis and, thus, it has been implicated in a vast number of conditions, notably cancer and neurodegeneration by ischemic damage, and the hope of therapeutic targets lies within [2]. Induction of ferroptosis is emerging as a prominent strategy in cancer treatment because it can attack a unique vulnerability in cancer cells, especially those that are resistant to traditional treatments like apoptosis. By inducing an iron-catalyzed form of cell death with lethal lipid peroxidation, ferroptosis not only kills tumor cells outright but also re-sensitizes resistant tumors to other treatments [3]. Furthermore, induction of ferroptosis can also remodel the tumor microenvironment to enhance anti-tumor immunity, complementing radiotherapy and immunotherapy. This dual benefit of bypassing drug resistance and enhancing immune-mediated cancer destruction makes induction of ferroptosis a promising avenue for more effective and tailored cancer therapies [4]. Mutations in cancer cells accumulate over time, but only a small subset, known as driver mutations, actively contribute to cancer progression, while the majority are neutral passenger mutations. Identifying these drivers is challenging due to tumor heterogeneity and the lack of a universal benchmark for validation. Various computational methods, including statistical models, functional impact analyses, and machine-learning techniques, have been developed to distinguish driver mutations based on mutation frequency, sequence context, and mutational signatures [5]. Mutations in cancer drive cell survival by disrupting the normal regulatory mechanisms that control cell growth and death. Driver mutations can continuously activate oncogenes or inactivate tumor suppressor genes, resulting in unchecked cell proliferation and resistance to apoptosis [6]. This genetic reprogramming enables cancer cells to bypass the usual cellular checkpoints, adapt to stress, and survive even under adverse conditions such as limited nutrients or therapeutic interventions. Furthermore, the accumulation of additional mutations contributes to tumor heterogeneity, allowing subsets of cells to evolve resistance to treatments and further promoting cancer cell survival and progression [7]. Identifying mutations that contribute to ferroptosis resistance is crucial for advancing cancer therapies. By pinpointing these genetic alterations, researchers can develop targeted strategies to sensitize cancer cells harboring specific mutations, making them more vulnerable to ferroptosis-inducing agents. This approach not only paves the way for precision medicine but also aids in recognizing treatments that are inherently effective in triggering ferroptosis in sensitive tumor profiles. Ultimately, this line of investigation holds promise for improving therapeutic outcomes and overcoming resistance in various cancer types [8]. This study aims to identify mutations associated with both ferroptosis resistance and sensitivity in various cancer cells, as well as to develop novel strategies to reverse these mechanisms.

## Ferroptosis pathways in cancer

**Fig. 1:**
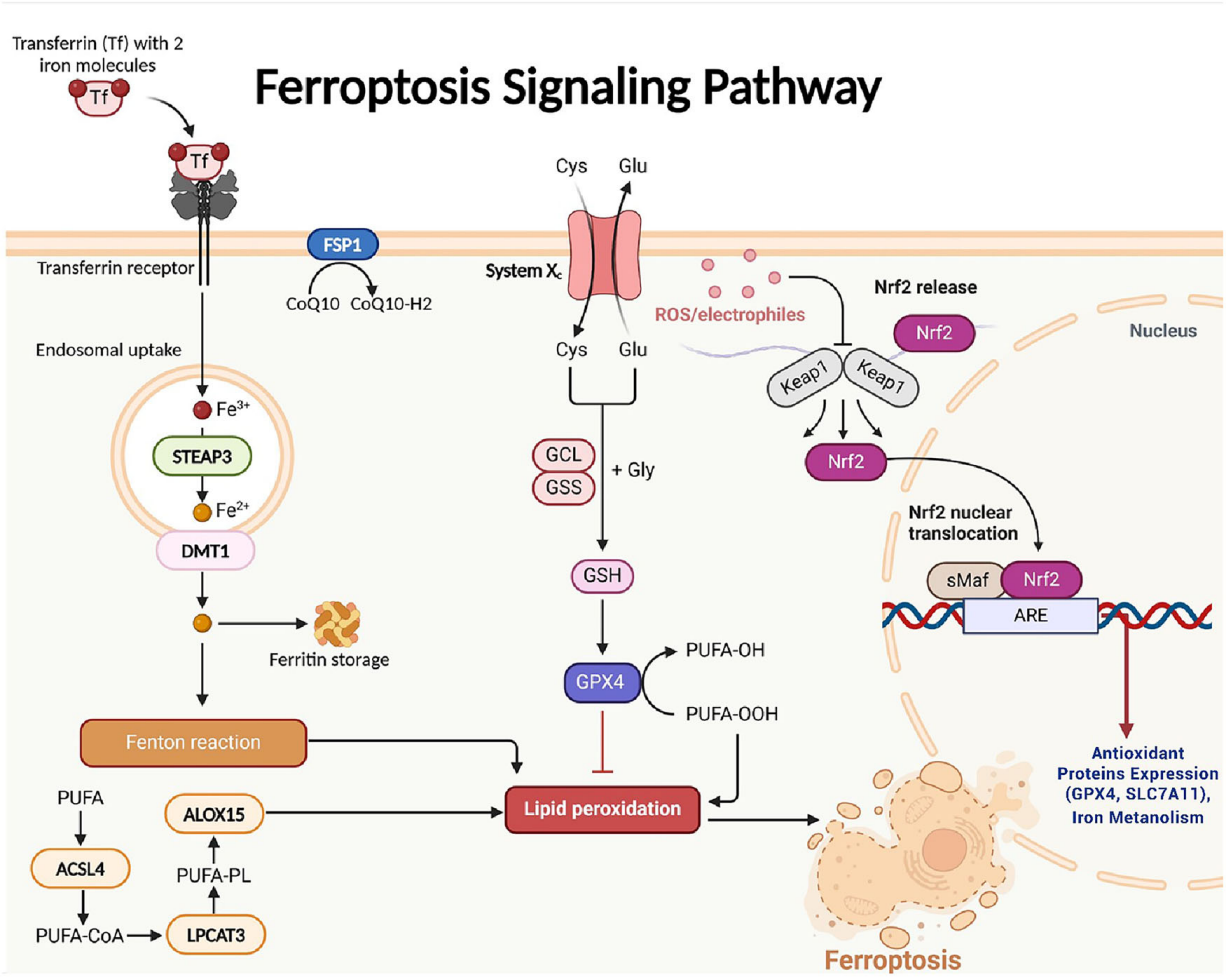
Comprehensive schematic of the ferroptosis signaling pathway in cancer. The diagram illustrates the iron-dependent lipid peroxidation process central to ferroptosis. Iron uptake occurs through transferrin (Tf) bound to the TfR1, followed by endosomal internalization, reduction of ferric iron (Fe³⁺) to ferrous iron (Fe²⁺) by STEAP3, and cytosolic release via DMT1. Labile iron can be sequestered in ferritin for storage or liberated via ferritinophagy to promote ROS generation through the Fenton reaction. In parallel, the cystine/glutamate antiporter system Xc⁻ (composed of SLC7A11/xCT and SLC3A2) imports cystine, which is reduced to cysteine for synthesis of GSH. GSH serves as an essential cofactor for GPX4, which reduces phospholipid hydroperoxides (PUFA-OOH) to harmless alcohols, thereby suppressing lipid peroxidation and ferroptosis. Conversely, ACSL4 and LPCAT3 incorporate PUFAs into membrane phospholipids, rendering them vulnerable to peroxidation. Additional GPX4-independent protective mechanisms include the FSP1–coenzyme Q10 (CoQ10)–NAD(P)H axis, DHODH, and the NRF2 pathway. Under oxidative or electrophilic stress, Keap1 releases NRF2, enabling its nuclear translocation. There, NRF2 heterodimerizes with small Maf (sMaf) proteins and binds to antioxidant response elements (AREs) in target gene promoters, upregulating expression of antioxidant proteins (including GPX4 and SLC7A11), iron regulators, and other cytoprotective factors to counteract lipid peroxidation. When these antioxidant defenses are overwhelmed, accumulated lipid peroxides disrupt membrane integrity, triggering ferroptotic cell death. This pathway holds substantial promise for targeted cancer therapies by exploiting ferroptosis vulnerabilities in resistant tumors.

Ferroptosis, a unique iron-dependent cell death mechanism driven by lipid peroxidation, stands apart from apoptosis and necroptosis. It is orchestrated by interconnected systems where metabolic pathways generate lipid substrates and reactive oxygen species (ROS) [9], and iron catalyzes oxidative reactions that damage cells [10]. Central to this process is glutathione peroxidase 4 (GPX4), which uses glutathione (GSH) to neutralize lipid peroxides, preventing cell death [11]. Additional protective mechanisms, including the ferroptosis suppressor protein 1 (FSP1)/CoQ10 axis, dihydroorotate dehydrogenase (DHODH), and GCH1/BH4 pathway, function independently of GPX4 to limit lipid oxidation, maintaining a delicate balance that determines cell survival or ferroptosis under oxidative stress and iron dysregulation [12]. The cystine/glutamate antiporter System Xc⁻, comprising xCT (SLC7A11) and SLC3A2, plays a critical role by importing cystine for GSH synthesis, a key antioxidant that mitigates lipid peroxidation. Overexpression of xCT in cancers promotes tumor survival by blocking ferroptosis, while its inhibition enhances ferroptosis and anticancer therapy efficacy [13] (Fig. 1).

GPX4 is pivotal, converting lipid hydroperoxides to non-toxic alcohols using GSH, preserving membrane integrity. Disruption of GPX4 through GSH depletion, small-molecule inhibition, or reduced expression escalates peroxidation, leading to membrane rupture and cell death, making it a prime target for overcoming drug-resistant tumors [14, 15]. Lipid metabolism drives ferroptosis, with polyunsaturated fatty acids (PUFAs) serving as primary peroxidation targets due to their double bonds. Acyl-CoA synthetase long-chain family member 4 (ACSL4) and lysophosphatidylcholine acyltransferase 3 (LPCAT3) facilitate PUFA integration, priming membranes for damage. Peroxidation, via lipoxygenases or free radicals, produces toxic peroxides that impair membrane function, countered by GPX4; imbalances trigger ferroptosis [16]. ALOX12 (12-lipoxygenase) oxygenates PUFAs, producing signals like 12-HETE, but mutations in its catalytic domain impair peroxide generation, reducing p53-mediated ferroptosis and aiding cancer cell survival [17]. Iron promotes ferroptosis through the Fenton reaction, in which labile ferrous iron catalyzes the generation of highly reactive oxygen species from hydrogen peroxide; these ROS subsequently initiate and propagate the peroxidation of PUFA-containing membrane phospholipids. Proteins such as transferrin, ferritin, and hepcidin maintain iron homeostasis, whereas ferritinophagy liberates stored iron, thereby expanding the labile iron pool and exacerbating oxidative lipid damage [18]. Iron fuels ferroptosis via the Fenton reaction, producing ROS that oxidize polyunsaturated fatty acids in membrane phospholipids. Iron homeostasis tightly regulates the labile iron pool: transferrin-bound Fe³⁺ is internalized by transferrin receptor (TfR1), reduced by STEAP3, released via divalent metal transporter 1 (DMT1), stored in ferritin, liberated through NCOA4-mediated ferritinophagy, and exported by ferroportin under hepcidin control [19]. Beyond the core GPX4-glutathione axis, GPX4-independent defenses prevent lipid peroxidation. Notably, FSP1 reduces coenzyme Q10 to ubiquinol via the FSP1–CoQ10–NAD(P)H pathway and vitamin K cycle, requiring N-myristoylation for membrane localization, a promising therapeutic target [20]. Mitochondrially, DHODH reduces ubiquinone to ubiquinol, scavenging radicals to inhibit ferroptosis [21]. The nuclear factor erythroid 2-related factor 2 (NRF2) pathway regulates ferroptosis by orchestrating antioxidant defenses and metabolic homeostasis. Under oxidative stress, NRF2 dissociates from Kelch-like ECH-associated protein 1 (Keap1), translocates to the nucleus, and induces genes promoting glutathione synthesis, iron sequestration, and lipid detoxification, thereby enhancing GPX4 and FSP1 to suppress peroxidation. In cancers, chronic NRF2 hyperactivity confers resistance to ferroptosis, underscoring its therapeutic potential [22, 23].

## Mutations in lung cancer associated with ferroptosis

**Fig. 2:**
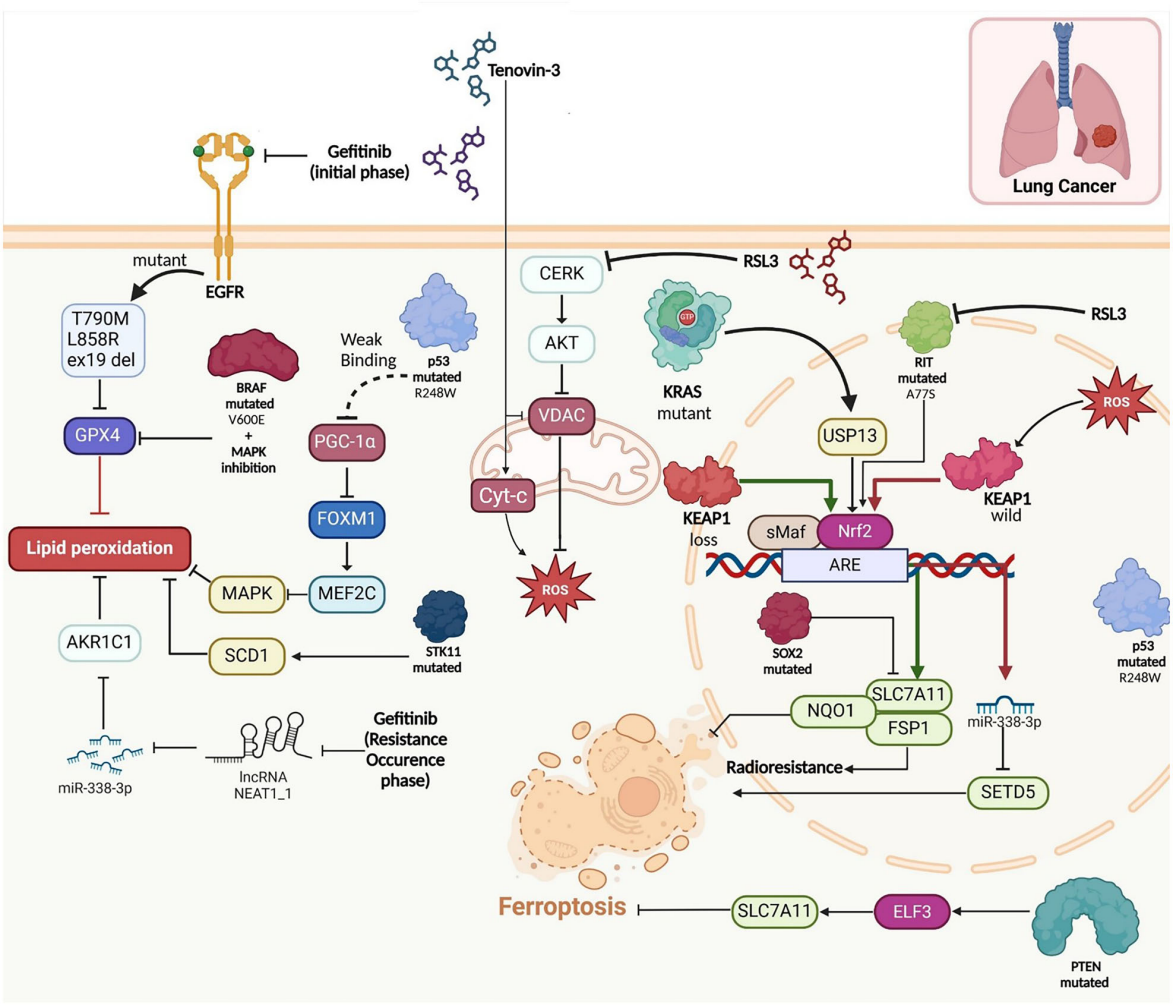
Schematic illustration depicting the influence of major genetic alterations in NSCLC on ferroptosis sensitivity and resistance, primarily through dysregulation of iron metabolism, lipid peroxidation, and antioxidant defense mechanisms. EGFR mutations promote ferroptosis vulnerability during initial treatment phases (such as with gefitinib) by suppressing GPX4 expression, disrupting iron balance, and modulating downstream lipid regulators like AKR1C1 and SCD1 via pathways involving MAPK and MEF2C. In contrast, KEAP1 loss or mutation results in constitutive activation of NRF2, which dimerizes with sMaf proteins at ARE to upregulate protective genes including NAD(P)H quinone dehydrogenase 1 (NQO1), thereby reinforcing antioxidant defenses and driving ferroptosis resistance. KRAS mutations (notably G12C) enhance ferroptosis susceptibility by inhibiting CERK, modulating USP13, and affecting voltage-dependent anion channel (VDAC)-mediated mitochondrial function, leading to cytochrome c (Cyt-c) release, elevated ROS, and increased vulnerability to inducers like RAS-selective lethal 3 (RSL3). TP53 mutations impair the peroxisome PGC-1α–forkhead box M1 (FOXM1) axis (with contributions from MEF2C), weakening oxidative stress regulation and ferroptosis control. PTEN mutations, often in concert with ELF3 transcription factor dysregulation, reprogram metabolism, such as by elevating SCD1 and solute carrier family 7 member 11 (SLC7A11, encoding the xCT subunit of system Xc⁻), to confer resistance to ferroptotic death. STK11 alterations similarly promote resistance through metabolic shifts. SOX2 mutations impair cystine uptake and can paradoxically increase ferroptosis sensitivity. Additionally, SETD5 contributes to radioresistance and ferroptosis suppression in certain contexts. Collectively, these mutation-specific pathways underscore the therapeutic promise of inducing ferroptosis (e.g., via agents like RSL3) to overcome treatment resistance and enhance precision strategies in genetically heterogeneous NSCLC.

Lung cancer, particularly non-small cell lung cancer (NSCLC), accounts for about 85% of all cases and is marked by a complex genetic landscape that influences its progression and treatment [24]. NSCLC is characterized by a complex landscape of gene mutations—including those in EGFR, ALK, BRAF, MET, KRAS, ROS1, and RET—that drive tumorigenesis through aberrant activation of signaling pathways such as MAPK, PI3K/AKT, and JAK/STAT. These mutations not only determine the tumor’s sensitivity to targeted therapies like tyrosine kinase inhibitors and monoclonal antibodies but also contribute to intrinsic and acquired resistance mechanisms [25]. This section reviews the effects of different gene mutations with ferroptosis resistance/sensitivity in lung cancer (Table 1) (Fig. 2).

**Table 1:** List of gene mutations associated with ferroptosis sensetivity/resistance.

Gene mutations	Location of mutation	Cell Line	Model (In vivo/In vitro)	Effect of mutation on ferroptosis	Ref.
**Lung cancer**					
** EGFR T790M/L858R**	Tyrosine kinase domain (T790M in exon 20; L858R in exon 21)	H1975	In vitro	Exhibits higher sensitivity to ferroptosis inducers (e.g., erastin) and mTOR inhibition (RAD001), suggesting a greater propensity toward ferroptosis induction.	[27]
** EGFR L858R**	Tyrosine kinase domain (L858R in exon 21)	H3255	In vitro	Shows relatively lower sensitivity to ferroptosis induction compared to resistant mutants; less responsive to mTOR inhibition-induced ferroptosis.	
** KRAS G12S (with wild-type EGFR)**	KRAS gene, codon 12 mutation (G12S)	A549	In vitro	Although EGFR is wild type, the presence of KRAS G12S correlates with an increased sensitivity to mTOR inhibitor-induced ferroptosis, similar to EGFR-resistant cells.	
** EGFR exon 19 deletion**	Exon 19 of the EGFR gene	PC9	In vitro	High sensitivity – tenovin-3 markedly induces apoptosis and ferroptosis in these cells.	[28]
** EGFR L858R mutation**	Typically in exon 21 of EGFR	NCI-H1975	In vitro	Lower sensitivity – tenovin-3 induces less cell death (ferroptosis/apoptosis) compared with PC9 cells.	
** EGFR E746-A750 deletion**	Variant within exon 19 of EGFR	NCI-H8827, NCI-H1650	In vitro	Reduced sensitivity – these cell lines show less response to tenovin-3 relative to PC9 cells.	
** EGFR wild type**	No mutation present	NCI-H1299, A549	In vitro	Minimal response – tenovin-3’s effects are less pronounced in these non-mutated cells.	
** EGFR (activating mutations)**	Exon 19 deletion or Exon 21 L858R – canonical mutations in LUAD	PC9, HCC827	In vitro and In vivo	In cells harboring these mutations, gefitinib can induce ferroptosis; however, sensitive cells undergo ferroptosis when treated, whereas resistant cells evade it.	[29]
** EGFR mutation**	Exon 19 deletion	PC9	In vitro	Less sensitive to cisplatin alone; ferroptosis significantly increased when combined with the inducer RSL3	[31]
** RIT1 A77S**	Switch II region (Ala 77 → Ser)	HBE135-E6E7 (stable overexpression)	In vitro	Increases susceptibility to ferroptosis induction by RSL3	[48]
** RIT1 F82L**	Switch II region (Phe 82 → Leu)	HBE135-E6E7 (stable overexpression)	In vitro	Increases sensitivity to RSL3-induced ferroptosis	
** RIT1 M90I**	Switch II region (Met 90 → Ile)	HBE135-E6E7 (stable overexpression); NCI-H2110 (endogenous mutation)	In vitro and In vivo (xenograft models)	Enhances ferroptosis induction (cells show increased RSL3/IKE-triggered cell death; knockdown reduces this effect)	
** KRAS (G12 mutations: G12A, G12C, G12D)**	Codon 12 of the KRAS gene (in lung adenocarcinoma patients)	Mutant KRAS: A549 (adenocarcinoma), H358 (bronchioloalveolar carcinoma), H460 (large cell carcinoma), H1792 (adenocarcinoma); Wild-type controls: H838, H1299	In vitro (cell survival, clonogenic assays, ROS/MMP measurements) and in vivo (mouse xenograft models)	In mutant KRAS cells, inhibition or downregulation of CERK leads to increased mitochondrial membrane potential (MMP), elevated ROS production, enhanced lipid peroxidation, and ultimately triggers ferroptotic cell death. This effect also sensitizes these cells to cisplatin treatment.	[34]
** KRAS^G12S**	Codon 12 (Glycine → Serine)	A549, H460 (KRAS mutant)	In vitro (cell culture) and in vivo (xenograft mouse model)	Activates KRAS signaling, which in turn upregulates USP13. USP13 stabilizes NFE2L2 (by reducing its K63-linked ubiquitination), promoting autophagy and thereby suppressing ferroptosis. Conversely, depletion or inhibition of USP13 shifts the balance—reducing NFE2L2 activity, lowering autophagy, and promoting ferroptosis.	[35]
** KRAS G12D and LKB1 loss (KL model)**	KRAS: G12D mutation in exon 2; LKB1: loss-of-function mutation (deleted alleles)	KL tumor-derived cell lines (TDCLs)	In vitro (TDCLs), In vivo (allograft, GEMM, PDX)	Combination of HCQ (autophagy inhibitor) and Trametinib (MEK inhibitor) markedly induces ferroptosis via mitochondrial dysfunction (reduced membrane potential, ATP production, and increased lipid peroxidation)	[131]
** KEAP1 Mutation/Knockout**	Occurs in lung cancer cells (commonly seen in non-small cell lung cancers)	Lung cancer cell lines (LUAD, LUSC models)	In vitro (cell cultures) and in vivo (xenograft models)	Loss of KEAP1 leads to NRF2 stabilization, which transcriptionally upregulates targets (e.g., FSP1, SLC7A11). This results in enhanced ferroptosis resistance and increased radioresistance.	[43]
** KEAP1 deletion/mutation**	Inactivation of KEAP1 (e.g., KO or mutation)	H1299, H23, A549, H460	In vitro (cell lines) and in vivo (xenografts)	Stabilizes NRF2, which upregulates FSP1; confers resistance to ferroptosis induced by class 1 (erastin) and class 2 (RSL3/ML162) FINs while not affecting class 3 (FIN56) response.	[42]
** KEAP1 G333C mutation**	Point mutation at position G333 in KEAP1	KEAP1-knockout FaDu cells reconstituted with mutant	In vitro	Reconstitution with KEAP1 G333C does not change NQO1 levels or alter ferroptosis induced by erastin/RSL3 compared to KEAP1 knockout alone (minimal impact).	[44]
** KEAP1 Knockout (Loss-of-function)**	Entire gene inactivation	FaDu (HNSCC)	In vitro	Increases NRF2 activation, upregulates NQO1 and SLC7A11, leading to resistance to class II ferroptosis inducers (RSL3, ML162) while not affecting sensitivity to FIN56.	
** STK11 Loss-of-function**	Distributed loss-of-function mutations (various regions within the gene; not confined to a single hotspot)	H358, H292 isogenic clones	In vitro	Promotes protection against ferroptosis (cells show partial resistance to ferroptosis inducers such as RSL3 compared to wild-type cells)	[45]
** KEAP1 Loss-of-function**	Distributed loss-of-function mutations (multiple regions; often accompanied by LOH on chromosome 19)	H358, A549, and other LUAD cell lines	In vitro and in vivo (xenograft models)	Increases NRF2 activity, leading to upregulation of antioxidant and ferroptosis-protective genes (e.g., SLC7A11, GCLC) that confer resistance to ferroptosis-inducing agents	
** STK11 and KEAP1 Co-mutation**	Combined loss-of-function mutations in both genes (frequently accompanied by loss-of-heterozygosity)	H358, H292, A549, H460 isogenic clones and patient-derived xenografts	Both in vitro and in vivo (xenograft models)	Synergistically enhances ferroptosis protection by markedly upregulating ferroptosis-protective gene networks (including SCD1 and AKR1C family members), creating a selective SCD1 dependency	
**Hematological malignancies**					
** ELF3 Overexpression** **+** **PTEN Deletion**	Combined genetic alteration in lung epithelium (Rosa26 knock-in + PTEN floxed deletion)	NL20 PTEN^–/–^ELF3ov; H1650 PTEN-nullELF3ov	In vivo (Ptend/dELF3OV/+ mice); In vitro	Inhibits ferroptosis via upregulation of SLC7A11, thereby promoting tumor development	[49]
** BRAFV600E**	BRAF gene, codon 600 (driver mutation)	HCC364, 1D, 1E parental cells	In vitro (with in vivo PDX data)	In the drug-tolerant persister (DTP) state, cells show increased sensitivity to GPX4 inhibitors (e.g., RSL3) that trigger ferroptosis. Vulnerability may be lost upon acquisition of further resistance.	[51]
** NRAS Q61K**	NRAS gene, codon 61 (activating mutation)	DFCI471 (patient biopsy-derived) and engineered HCC364NRAS	In vitro	MAPK reactivation via NRAS Q61K increases antioxidant defenses (e.g., upregulation of SLC7A11), rendering cells resistant to ferroptosis inducers such as RSL3 and erastin.	
** EGFR amplification**	Amplification of EGFR gene region	HCC364 D/T-resistant clone (HCC364^EGFRa)	In vitro	EGFR amplification leads to sustained MAPK signaling and enhanced antioxidant responses, reducing sensitivity to ferroptosis triggers.	
** BRAF amplification**	Amplification of the BRAF gene region (CNV increase from ~2 to ~15.4)	1E-R cells (PDX-derived D/T-resistant cells)	In vitro and in vivo (xenografts)	BRAF gene amplification reactivates MAPK signaling. This is accompanied by increased SLC7A11 expression and cystine uptake, thereby decreasing ferroptosis sensitivity while creating a vulnerability to HDAC inhibitors.	
** SLC7A11 promoter mutation**	Disruption of the SOX2 binding motif (the conserved “CTTTGTT” site) in the SLC7A11 promoter	H1299 (also studied in SW620 and HCT116 oncosphere models)	In vitro lung cancer cell models (including oncosphere culture used to model cancer stem-like cells)	Reduced SLC7A11 expression, leading to lower intracellular cysteine/GSH levels and increased sensitivity to ferroptosis (induced by erastin/IKE).	[55]
** SOX2 C265S Mutation**	Cys265 in the C-terminal transactivation domain of SOX2	H1299 and HEK293T (for luciferase reporter assays)	In vitro cell line assays assessing transcriptional activity and ferroptosis sensitivity	Mutation (Cys265 to Ser) prevents the oxidation of SOX2. This results in enhanced SOX2 transcriptional activity, higher SLC7A11 expression, and thus increased resistance to ferroptosis	[47]
** TP53 R248W mutation**	DNA-binding domain (R248W hotspot)	CALU-1, NCI-H358	In vitro (cell culture) and In vivo (xenograft mouse model)	Confers resistance to ferroptosis; mutant TP53 shows weak binding with PGC-1α leading to elevated FOXM1/MEF2C expression, reduced lipid ROS accumulation, and enhanced cell survival under ferroptotic stress.	
** Other TP53 mutations (e.g., R175H, R273H/L)**	DNA-binding domain (hotspot mutations)	Detected in patient tissue samples	Clinical tissue analysis	Associated with malignant progression in early lung cancer; while not directly modeled in cell lines here, these mutations are implied to contribute to ferroptosis insensitivity similar to R248W.	[47]
** TP53 mutant (R175H)**	TP53 gene, codon 175 (DNA-binding domain alteration)	CALU-1, NCI-H358	In vitro (cell experiments) and in vivo (mouse tumor model)	Inhibits ferroptosis (lower levels of MDA, ferrous ions, and ROS, with enhanced cell survival)	[46]
** STK11 loss-of-function mutation**	Not explicitly detailed (general inactivation of STK11)	A549, H23, H2030 (naturally mutated cells)	In vitro	Suppresses ferroptosis by increasing expression of ferroptosis inhibitors (e.g., SLC7A11) and promoting MUFA synthesis	[52]
** FLT3-ITD**	Juxtamembrane domain (internal tandem duplication)	MOLM-14, MV4-11	In vitro; Patient-derived xenografts (in vivo)	Drives high lipogenic activity via C/EBPα. FLT3 inhibition reduces MUFA production, increasing the PUFA/MUFA ratio and sensitizing cells to ferroptosis.	[64]
** FLT3-TKD**	Tyrosine kinase domain	AML cell lines	In vitro; Patient samples (in vivo context)	Present in a subset of AML; does not markedly alter ferroptosis response compared to FLT3-ITD when treated with FLT3 inhibitors.	
** GADD45A deletion**	Not specified (loss-of-function alteration)	AML leukemia stem cells (murine models, THP-1 cells, PDX cells)	Both in vivo (mouse models, serial transplantation) and in vitro (cell culture assays)	Loss of GADD45A leads to reduced ROS levels and upregulation of FTH1-mediated antioxidant defense, thereby conferring resistance to ferroptosis.	[65]
** DDX11 missense mutation**	Exon 21 (plus several synonymous changes in exons 19–26)	AML LSCs isolated from murine models	In vivo (observed in serial transplantation models)	Induces replication stress and genomic instability. These alterations may help cells adapt to stress conditions and indirectly contribute to resistance against ferroptosis.	
** HOXA cluster mutations (HOXA4, HOXA5, HOXA9, HOXA10)**	Coding regions (exact loci not detailed)	AML LSCs from murine AML models	In vivo (serial transplantation experiments)	Mutations in HOXA genes promote enhanced self-renewal and stemness. This supports low ROS levels and may indirectly decrease sensitivity to ferroptosis through sustained survival signaling.	
** DNMT1 mutation**	Not specified	AML LSCs from murine models	In vivo (mouse AML models)	Altered DNA methylation status due to DNMT1 mutations may reinforce GADD45A suppression, indirectly contributing to the antioxidant state and ferroptosis resistance observed in these cells.	
** TP53 p.C176G (missense)**	Exon 5 (DNA binding domain)	OCI-LY1	In vitro	APR-246 induces ferroptosis effectively with a low IC50, indicating high sensitivity.	[67]
** TP53 p.G245D (missense)**	Exon 7 (DNA binding domain)	OCI-LY7	In vitro and In vivo (xenograft in mice)	APR-246 triggers both ferroptosis and p53-dependent ferritinophagy (autophagy related to ferritin degradation), resulting in robust cell death.	
** TP53 Wild-Type**	–	OCI-LY3	In vitro	APR-246 induces ferroptosis through mechanisms that are partially dependent on p53 and partially independent.	
** TP53 nonsense mutation**	–	TMD8	In vitro	APR-246 induces ferroptosis, with sensitivity comparable to TP53 wild-type cell lines.	
** TP53 splice mutation**	–	SU-DHL-6, SU-DHL-10, SU-DHL-16	In vitro	These cell lines exhibit lower sensitivity (i.e., higher IC50) to APR-246 and show weaker induction of ferroptosis.	
** NPM1c (NPM1 mutation)**	C-terminal region (not detailed in the study)	OCI-AML3 and IMS-M2 cell lines; primary AML samples	In vitro (cell lines/ex vivo) and in vivo (PDX)	NPM1-mutated cells are more sensitive to xCT inhibition. SSZ induces ROS accumulation and lipid peroxidation; ferroptosis inhibitors partially rescue viability, suggesting a partial role of ferroptosis.	[68]
** FLT3-ITD, DNMT3AR882H, IDH1R132H (in combination with NPM1c)**	FLT3-ITD: typically juxtamembrane; DNMT3AR882H: catalytic domain; IDH1R132H: active site (not specified in study)	Primary AML cells (patient-derived xenograft)	In vivo (PDX model)	xCT inhibition triggers ROS-mediated cell death. Partial rescue by ferroptosis inhibitors (ferrostatin-1 and deferoxamine) indicates ferroptosis contributes to cell death.	
** CEBPA, RUNX1, ASXL1, EZH2, TET2, JAK2**	Not specified	Primary AML cells (patient-derived xenograft)	In vivo (PDX model)	While the study focused on xCT inhibition effects overall, specific ferroptosis assays were not reported for these mutations. The ROS-dependent cell death mechanism is assumed to be similar.	
**Liver cancer**					
** CTNNB1 gain-of-function (GOF) mutation**	Typically in the N-terminal region (Exon 3)*	Human HCC samples; cell lines such as HepG2, Huh6, SNU398	In vitro (cell culture experiments) and in vivo (mouse models, xenograft studies, hydrodynamic tail vein injection models)	Active CTNNB1 mutation upregulates SLC13A3 expression, which increases GSH uptake and modulates SLC7A5-mediated leucine transport. Inhibition of SLC13A3 induces autophagic ferroptosis in β-catenin–activated liver cancer cells.	[71]
** LOXL3 S704 Mutants (S704A and S704D)**	Serine 704 in LOXL3, a critical phosphorylation site within the mitochondrial region	Human liver cancer cell lines (e.g. Huh7 and Hep3B) and genetically engineered mouse models (S704D knock-in)	In vitro (cell culture assays with Oxaliplatin treatment) and in vivo (xenograft models and SB transposon-induced liver cancer models)	The phospho-mimic mutant (S704D) increases LOXL3 lysyl-oxidase activity, which stabilizes DHODH by preventing its ubiquitination at K344, thereby reducing lipid peroxidation and ferroptosis and promoting chemoresistance. In contrast, the dephosphorylation mimic (S704A) fails to protect DHODH, leading to increased mitochondrial lipid peroxidation and enhanced ferroptosis under chemotherapy.	[73]
** DHODH K344 mutation (K344R)**	Lysine 344 in DHODH, the key residue for ubiquitination vs. oxidation	Human liver cancer cell lines (e.g., Huh7 and Hep3B)	In vitro (transfection experiments under Oxaliplatin treatment)	The K344R mutation prevents ubiquitination of DHODH at lysine 344, resulting in stabilized DHODH protein levels, reduced lipid peroxidation, and diminished ferroptosis, thereby contributing to increased chemoresistance.	
** AK2 mitochondrial signal peptide mutant (K14G/R17G)**	Within the mitochondrial targeting region of AK2 (affecting its mitochondrial localization)	Human liver cancer cell lines (e.g., Huh7 and Hep3B)	In vitro (cells with AK2 knockdown and rescue with mutant vs. wild-type AK2)	Disruption of AK2’s mitochondrial localization (via the K14G/R17G mutation) leads to decreased phosphorylation of LOXL3 at S704. This results in lower LOXL3 activity in mitochondria, increased lipid peroxidation, and enhanced ferroptosis under chemotherapy conditions.	
** TP53 mutant**	Not specified (mutation present)	PLC/PRF/5	In vitro	Altered p53 function in mutant cells leads to an intermediate response; the dysregulated p53 signaling in PLC/PRF/5 cells results in a less robust induction of ferroptosis.	[74]
** BAP1 somatic mutation**	Various mutations (splicing, nonsense, missense, and frameshift) distributed across multiple protein domains (e.g., G45R, M115Rfs*9, Q156*, etc.)	Clinical HCC samples from TCGA and tissue microarrays	In silico analyses and clinical correlation studies (in vivo)	HCC cases harboring BAP1 mutations exhibit significantly lower BAP1 mRNA/protein expression compared to wild-type cases. These mutations are associated with a reduced capacity to promote ferroptosis, as reflected by the altered expression of ferroptosis-related genes (with decreased levels of factors such as ALOX15, FDFT1, NCOA4, and DPP4), while high BAP1 expression correlates with enhanced ferroptosis.	[76]
** CP frameshift mutation (c.1192-1196del, p.Leu398Serfs)**	Novel frameshift mutation in CP gene	CP knockdown rescue in HepG2 and Hep3B	In vitro	Fails to rescue CP depletion–enhanced ferroptosis; the mutant form is unable to regulate iron homeostasis, resulting in persistent iron accumulation and elevated lipid ROS despite overexpression.	[77]
Colorectal cancer					
** KRASG12D mutation with MRTX1133 treatment**	KRAS gene (codon 12) – mutated background	LS180-KRASG12D, LS174T-KRASG12D	In vitro*	MRTX1133 treatment in these KRASG12D-mutated cells increases ferroptosis markers (total iron, ferrous iron, lipid ROS, MDA, GSSG/GSH ratio).	[81]
** KRAS mutation**	KRAS gene – inherent oncogenic mutation (e.g., codon 12 alterations)	HCT 116, LoVo, SW620	In vitro; In vivo (xenograft using HCT 116)	Provides the oncogenic context; these KRAS-mutant cells are susceptible to ferroptosis when treated with TYM-3–98.	[82]
** p53 deletion**	Complete loss (null mutation)	HCT-116 (p53 wild-type vs. p53-null)	In vitro	Nearly complete suppression of basal ferroptosis (protection against ferroptosis)	[80]
** KRAS G12D**	Codon 12 (G12D mutation)	SW48 (parent vs. KRAS mutant)	In vitro	Minimal impact on basal ferroptosis despite increased SLC7A11 expression	
** p53 mutation (e.g., R273H, P309S)**	DNA binding domain (commonly exon 7)	HT29 and SW480	In vitro and xenograft in vivo	p53-mutant colon cancer cells are intrinsically resistant to ferroptosis. In these cells, high MDM4 levels further inhibit ferroptosis by stabilizing GPX4 via TRIM21-mediated ubiquitination switching (from degradative K48-linked to stabilizing K63-linked), reducing lipid peroxidation and enhancing chemoresistance.	[83]
** AMER1 truncating (nonsense) mutations**	Hotspot regions in interaction domains (aa 452–571, 768–830, 831–907)	HCT8, SW480, HCT116, DLD1 (CRC)	In vitro & In vivo	Loss of AMER1 function leads to reduced ubiquitination/degradation of SLC7A11 and FTL. This results in increased cystine uptake, elevated GSH synthesis, decreased labile iron, and ultimately resistance to ferroptosis, thereby promoting hematogenous metastasis.	[84]
** AMER1 loss-of-function mutations in other cancers**	Similar regions affecting binding domains	AGS (gastric cancer); SK-MEL-2 (melanoma)	In vitro	AMER1 deficiency causes upregulation of SLC7A11 and FTL, contributing to a resistance phenotype against erastin-induced ferroptosis.	
** SLC7A11 degron motif mutation**	β-TrCP degron motif (key serine residue mutated to alanine)	CRC cell lines (e.g., SW480)	In vitro	Mutation in the degron motif reduces K48-linked ubiquitination of SLC7A11, leading to its stabilization and decreased lipid peroxidation, thus impairing ferroptosis induction.	
** HECTD3 C823A mutation**	Ubiquitinase active site (Cysteine 823)	SW480, HCT116, 293 T cells	In vitro (cell assays) and in vivo (xenograft models)	Impairs polyubiquitination of SLC7A11, leading to its stabilization. This results in enhanced cystine uptake, increased glutathione synthesis, and resistance to ferroptosis, which promotes tumor growth.	[85]
**HECTD3 C823A mutation**	Ubiquitinase active site (Cysteine 823)	SW480, HCT116, 293 T cells	In vitro (cell assays) and in vivo (xenograft models)	Impairs polyubiquitination of SLC7A11, leading to its stabilization. This results in enhanced cystine uptake, increased glutathione synthesis, and resistance to ferroptosis, which promotes tumor growth.	[87]
Pancreatic cancer					
** KRAS mutation (e.g., KRASG12D/G12V)**	Exon 2 (e.g., codon 12 mutations)	CFPAC-1, PANC-1	In vitro and In vivo	Contributes to ferroptosis resistance by upregulating downstream targets (including TMOD3), which helps cancer cells evade ferroptosis-inducing stimuli.	[132]
** KRASG12D mutation**	Mutation at codon 12 in the KRAS gene	Parental PDAC cells (AsPC1, KPC210)	In vitro & in vivo xenograft	Drives oncogenic signaling; under MRTX1133 treatment, parental cells undergo ferroptosis leading to tumor regression	
** KRAS and TP53 mutation**	Pancreatic ductal adenocarcinoma tissue (PDAC)	PANC1, MiaPACA2 (human PDAC); KPC (mouse PDAC)	In vitro cell cultures and genetically engineered mouse models (GEMMs)	N6F11 triggers selective ferroptosis by promoting TRIM25-mediated GPX4 degradation, leading to increased lipid peroxidation and cell death in cancer cells while sparing immune cells.	[133]
** KRAS mutation**	Not specified (commonly codon 12)	AsPC-1	In vitro and in vivo	KRAS mutant cells display sensitivity to artesunate-induced ferroptosis; this sensitivity is further enhanced when GRP78 is inhibited, leading to reduced cell viability and tumor growth.	[104]
** KRAS mutation**	Not specified (commonly codon 12)	PaTU8988	In vitro	Similar to AsPC-1, PaTU8988 cells exhibit artesunate-induced ferroptosis, highlighting that KRAS mutation contributes to metabolic dependencies that favor ferroptotic cell death.	
**Breast cancer**					
** BRCA1 (Brca1(L63X/+)**	Nonsense mutation at codon 63 (early region of BRCA1)	Primary kidney cells from the rat; rat heterozygous knockout model	In vivo (rat model)	Promotes ferroptosis-resistance, reducing sensitivity to iron-induced cell death	[90]
** BRCA1 loss-of-function**	Endogenous mutation (BRCA1-deficient background; e.g., UWB1.289 cells)	UWB1.289 ovarian cancer cells; BRCA1-mutant ovarian cancer patient–derived organoids (BRCA1 c.3756_3759del)	In vitro (cell lines, PDO) & in vivo (xenografts)	Loss of BRCA1 elevates GPX4 protein levels, leading to resistance to ferroptosis and reduced lipid peroxidation.	[91]
** BRCA1 I26A mutation**	RING domain (N-terminal 1–303 region; I26A substitution)	A2780 cells stably expressing mutant BRCA1	In vitro (cell lines)	Impaired E3 ubiquitin ligase activity prevents GPX4 ubiquitination/degradation, resulting in decreased ferroptosis.	
** p53 R273H (mutant)**	DNA-binding domain	MDA-MB-468 breast cancer cells	In vitro	p53 reactivators (e.g., NSC-59984, Inauhzin, Coti-2) reduce CETZOLE-induced ferroptotic cell death, offering protection despite mutant p53 expression.	[134]
** p53 R280K (mutant)**	DNA-binding domain	MDA-MB-231 breast cancer cells	In vitro	p53 reactivators similarly protect these cells from ferroptosis triggered by CETZOLE, contrary to the expected sensitization effect.	
** p53 R273H (mutant)**	DNA-binding domain	MDA-MB-468 breast cancer cells	In vitro	p53 reactivators (e.g., NSC-59984, Inauhzin, Coti-2) reduce CETZOLE-induced ferroptotic cell death, offering protection despite mutant p53 expression.	[92]
** p53 R280K (mutant)**	DNA-binding domain	MDA-MB-231 breast cancer cells	In vitro	p53 reactivators similarly protect these cells from ferroptosis triggered by CETZOLE, contrary to the expected sensitization effect.	
** CISD2 H114C**	Mutation at histidine 114 in the [2Fe–2S] cluster binding domain	MDA-MB-231 human epithelial breast cancer cells	In vitro	Disruption of CISD2 function leads to increased mitochondrial labile iron (mLI) and ROS (mROS), upregulation of TXNIP, decreased GPX4 and TRX2, increased TfR expression and lipid peroxidation, triggering a ferroptosis-like cell death pathway	[93]
**Ovarian cancer**					
** p53 missense mutation**	Not specified	KURAMOCHI	In vitro (cell line studies)	Mutant p53 loses its normal function; MEX3A does not interact with mutant p53, suggesting that ferroptosis regulation via p53 is impaired.	[108]
**Glioma**					
** IDH1 R132C**	Exon 4 – Codon 132 (R132C)	HT-1080 (endogenous mutant); KYSE-170 (overexpression)	In vitro	Increases sensitivity to erastin-induced ferroptosis via production of D-2-hydroxyglutarate (2-HG), which leads to GPX4 downregulation, enhanced lipid ROS accumulation, and accelerated glutathione depletion.	[95]
** IDH1 R132C/T77A**	Exon 4 – Codon 132 (R132C) and Codon 77 (T77A)	HT-1080 (IDH1+/− reconstituted cells)	In vitro	Partially sensitizes cells to erastin-induced ferroptosis; however, due to impaired 2-HG production, the effect is reduced compared to the R132C mutation.	
** IDH1 R132H**	Codon 132 in the IDH1 gene (active site)	U87 and NHA cells engineered to express mutant IDH1; patient-derived BTICs (e.g., TS603, BT142)	In vitro (cell cultures) and in vivo (xenograft mouse models)	Activates the PI3K/AKT/Nrf2 axis, leading to upregulation of antioxidant genes (GCLC, GCLM, NQO1, SLC7A11) that protect against lipid peroxidation and ferroptosis. Inhibition of AKT reverses this protection, sensitizing cells to ferroptotic cell death.	[96]
Nrf2 phosphorylation mutant (Nrf2DD)*	Lacks critical motifs (DIDLID and SDS motif) required for b-TrCP–mediated ubiquitination (precise residues not detailed)	NHA cells in an IDH1-mutated background	In vitro	Disrupts the normal degradation of Nrf2; the phosphorylation-insensitive mutant fails to be ubiquitinated, which in experimental settings abolishes the chemosensitization effect by AKT inhibitors, thus altering ferroptosis regulation.	
** IDH1-R132H**	Codon 132 in the IDH1 gene (Arginine → Histidine)	Mouse renal tubular epithelial cells (TECs); primary proximal tubular cells (PTCs) isolated from Idh1WT/MutKspCre mice; 293 T cells used for reporter assays	Both in vivo (Idh1WT/MutKspCre mice treated with cisplatin) and in vitro (cultured TECs/PTCs)	Exacerbates cisplatin-induced mitochondrial lipid peroxidation and dysfunction, leading to increased ROS accumulation and sensitization of tubular epithelial cells to ferroptotic cell death. This mutation indirectly promotes ferroptosis by altering epigenetic regulation of mitochondrial genes.	[97]
** NDUFA1 promoter hypermethylation (epigenetic effect)**	Promoter region of NDUFA1 (chrX:37191032–37191910; covering ~74 CpG sites)	Primary PTCs from Idh1WT/MutKspCre mice; 293T cells (luciferase reporter assays); HK-2 cells (NDUFA1 knockout experiments)	Both in vivo (kidney tissues from mutant mice) and in vitro (cultured PTCs/HK-2 cells)	Leads to transcriptional repression of NDUFA1, resulting in decreased NDUFA1 protein levels. This disruption impairs the normal interaction between NDUFA1 and FSP1, hindering mitochondrial ROS clearance and thereby exacerbating cisplatin-induced ferroptosis in renal tubular cells.	
** p53 mutant (e.g., R273H, R213Q)**	p53 mutations in the DNA binding domain (e.g., R273H in U251, R213Q in U118)	U251 (p53 R273H), U118 (p53 R213Q)	In vitro (GBM cell lines) and validated in xenograft models	In p53-mutant GBM cells, p62 overexpression decreases SLC7A11 expression and increases lipid peroxidation, thereby promoting ferroptosis. The enhanced mutant-p53/NRF2 interaction by p62 leads to stronger suppression of NRF2’s antioxidant activity.	[98]
**Thyroid cancer**					
** BRAFV600E**	BRAF gene, codon 600 (exon 15)	BCPAP and IHH4 cells; Xenograft mouse models	In vitro (BCPAP, IHH4) and In vivo (Xenografts)	The BRAFV600E mutation leads to upregulation of ARSI, which suppresses EREG-mediated ferroptosis. This suppression results in increased resistance to ferroptosis and contributes to tumor progression and sorafenib resistance.	[135]
** BRAFV600E** **+** **PIK3CA co-mutation**	BRAFV600E: codon 600 in BRAF; PIK3CA: (co-mutation details not specified)	K1 cells (thyroid cancer cell line)	In vitro (monolayer and 3-D spheroids)	GPX4 inhibitors (RSL-3, ML-162) robustly induce ferroptosis—evidenced by increased ROS, GSH depletion, reduced proliferation and migration; notably, GPX4 protein levels decrease in monolayer K1 cells.	[106]
** BRAFV600E** **+** **TERT promoter mutation**	BRAFV600E: codon 600 in BRAF; TERT promoter mutation (location within promoter region)	MDA-T32 cells (thyroid cancer cell line)	In vitro (monolayer)	GPX4 inhibition causes a dramatic, dose-dependent increase in ROS (up to ~800%), marked DNA damage, and potent suppression of migration, indicating strong ferroptosis activation.	
** NRAS** **+** **TERT promoter mutation**	NRAS mutation (specific codon not detailed); TERT promoter mutation	MDA-T68 cells (thyroid cancer cell line)	In vitro (monolayer)	GPX4 inhibitors lead to significant ROS induction and GSH depletion; these cells show lower baseline GSH and high sensitivity to ferroptosis—evidenced by inhibited migration and distinct DNA damage responses.	
**Melanoma**					
** p53 E285K**	Located in the C-terminal helix of the DNA-binding domain (disrupts a salt-bridge network essential for proper DNA recognition)	Patient-derived melanoma sample (M#31 cells)	In vitro (monolayer cultures and 3D spheroids)	Mutp53(E285K) loses transcriptional activity and localizes predominantly to the cytosol, leading to resistance against cisplatin-induced cell death. However, this chemoresistance can be partially overcome by inducing ferroptosis (e.g., using GPX4 inhibitor RSL3 or AKT inhibition).	[136]
** p53 R175H/R248W (E285 cluster)**	Located within or adjacent to the DNA-binding domain; these mutations (including K132E in the same network) are predicted to have similar destabilizing effects as E285K	Reconstituted melanoma cells (e.g., in M#31 or M#54 models)	In vitro	These gain-of-function mutants also show impaired DNA binding and p53-dependent transcription, contributing to chemoresistance. Like E285K, their resistance to cisplatin is evident, and ferroptosis induction (via GPX4 inhibition) represents a potential strategy to counteract this resistance.	
**RCC**					
** DJ-1 T19A**	Threonine 19 in DJ-1 (O-GlcNAcylation site in the N-terminal region)	Renal cell carcinoma cell lines (e.g., 786-O, 769-P)	In vitro (with supporting in vivo xenograft data)	The T19A mutation prevents O-GlcNAcylation of DJ-1, destabilizing its homodimeric structure. This disruption promotes enhanced binding between SAHH and its inhibitor AHCYL1, impairs homocysteine synthesis in the transsulfuration pathway, lowers glutathione (GSH) levels, and thereby increases lipid ROS accumulation leading to enhanced ferroptosis.	[100]
** VHL mutation**	-	HK-2 cells, mRTCs, HEK293 cells	In vitro	Increased lipid ROS production, decreased GPX4 expression, ferroptosis induced via LCN-2 pathway	[101]
** KDM5C mutation**	KDM5C gene (X-inactivation escaping gene)	RCC4 (ccRCC cell line), ACHN, Caki-1, 769-P, A498	In vitro, In vivo (Kdm5c-/- mice)	Decreased ferroptosis resistance, increased glycogen accumulation, up-regulation of PPP. KDM5C depletion confers resistance to ferroptosis by enhancing PPP and NADPH production.	[103]
**Cervical cancer**					
** KDM4A K471R**	K471 (Lysine residue)	SiHa, Hela	In vitro	Weakened KDM4A-SUMO1 binding; decreased ferroptosis resistance in CC cells under hypoxia-like conditions.	[107]
**Gastric cancer**					
** CDH1 mutation (Loss of function)**	Whole gene (loss of expression)	SNU16, SNU668	In vitro, Xenograft	Loss of E-cadherin expression sensitizes DGC cells to ferroptosis, increasing sensitivity to ferroptosis inducers like RSL3 and erastin.	[137]
**Wilms tumor**					
** DROSHA mutation**	RNase III domains (Asp, Glu, Gln)	WiT49 (Wilms tumor)	In vitro	Mutation impairs microRNA production, leading to de-repression of target genes and enhanced sensitivity to ferroptosis.	[109]
** DICER1 mutation**	RNase III domains (similar to DROSHA)	WiT49 (Wilms tumor)	In vitro	Similar to DROSHA mutations, DICER1 mutations impair microRNA processing, leading to an increased ferroptosis sensitivity.	[105]
**Cholangiocarcinoma**					
** KRAS** **+** **BAP1 mutation**	KRASG12D with BAP1 homozygous deletion	Liver (albumin-expressing cells)	In vivo (GEMM)	Combined loss of BAP1 and KRAS mutation (BhomoKA) is required for ICC development, with altered ferroptosis regulation as a contributing mechanism. Increased SLC7A11 expression observed.	[138]
** NF2 mutation**	Chromosome 22q12.2 (Somatic mutation, allelic loss)	CH157 (NF2 mutant), IOMM-Lee (NF2 intact), PM3 (NF2 mutant)	In vitro (cell lines), In vivo (mouse xenografts)	Increased sensitivity to ferroptosis. NF2 inactivation leads to higher susceptibility to Erastin-induced ferroptosis, indicated by increased lipid peroxidation (MDA) and cytotoxicity (LDH release)	
** E-cadherin Loss**	N/A (Cell density-dependent regulation)	IOMM-Lee, CH157, PM3	In vitro (cell lines)	Increased sensitivity to ferroptosis. Knockdown of E-cadherin at high cell density promotes lipid peroxidation and cell death, independent of NF2 status	

### EGFR

EGFR mutations are pivotal in NSCLC, driving aberrant protein activation that promotes tumor growth, survival, and metastasis. These alterations serve as biomarkers for TKI responsiveness. Common variants like exon 19 deletions and L858R predict TKI sensitivity, but co-occurring T790M mutations confer resistance, impacting outcomes [26]. EGFR mutations significantly influence ferroptosis susceptibility. Cells with resistant T790M/L858R mutations show heightened sensitivity to ferroptosis inducers compared to those with L858R alone, due to lower GPX4 levels and altered iron metabolism proteins like FTH1 and ferroportin. This reduces their ability to counter reactive oxygen species (ROS) and lipid peroxides under oxidative stress, increasing ferroptotic cell death [27]. In TKI-resistant NSCLC with EGFR Exon 19 deletion (ex19 del), disrupted signaling fosters redox dysregulation and oxidative stress, heightening ferroptosis vulnerability. This positions ferroptosis inducers as key to surmounting resistance to apoptosis-targeted therapies. Notably, tenovin-3 elicits combined apoptotic-ferroptotic cell death in ex19 del models, offering a resistance-overcoming paradigm [28]. In EGFR-mutated LUAD acquiring resistance to EGFR-TKIs like gefitinib, which primarily induces apoptosis via EGFR inhibition, upregulated aldo-keto reductase family 1 member C1 (AKR1C1) detoxifies lipid peroxides, mitigating ferroptotic effects. This resistance is driven by NEAT1_1, which sponges miR-338-3p to boost AKR1C1, highlighting adaptive ferroptosis defenses in persistent cells [29]. Osimertinib-tolerant EGFR-mutant cells exhibit ferroptosis vulnerability due to metabolic reprogramming and iron dysregulation, accumulating lipid peroxides and ROS. Elevated ferrous ions fuel the Fenton reaction, and ferroptosis inducers like RSL3, which inhibit GPX4, overwhelm defenses, causing cell death. Iron chelation with deferoxamine confirms iron’s role by restoring viability [30]. EGFR ex19 del NSCLC cells, PC9, show reduced susceptibility to cisplatin-induced ferroptosis versus A549 wild-type, unlike their general ferroptosis vulnerability. Combining cisplatin with RSL3 amplifies ferroptosis in mutants via heightened lipid peroxidation. This triggers immunogenic cell death, activating neutrophils and boosting anti-tumor immunity, countering checkpoint inhibitor resistance [31].

### Kras

KRAS-mutant lung cancers, particularly KRASG12C, are challenging due to the protein’s strong GTP affinity, historically deemed undruggable [32]. Early studies suggested RAS-mutant cells’ sensitivity to ferroptosis, but recent findings indicate this is not always sufficient. CRISPR data highlight vulnerabilities in genes like GPX4, ACSL3, and FASN in KRAS- and NRAS-mutant NSCLC [33]. Inhibiting ceramide kinase (CERK) in KRAS-mutant NSCLC disrupts ceramide-1-phosphate, impairing AKT-mediated VDAC phosphorylation, destabilizing mitochondria, and increasing ROS, thus triggering ferroptosis. This sensitizes cells to cisplatin, suggesting a synergistic strategy [34]. In KRAS-mutated lung adenocarcinoma, deubiquitinase ubiquitin-specific peptidase 13 (USP13) exemplifies autophagy-ferroptosis crosstalk by stabilizing NRF2, upregulating SQSTM1/p62, and SLC7A11 to favor autophagy and suppress ferroptosis [35]. Other pathways, like NCOA4-mediated ferritinophagy, promote ferroptosis via iron liberation. USP13 inhibition shifts this toward lipid peroxidation and cell death [36]. In KRAS-mutant bone metastasis, high HOXC10 activates NOD1/ERK, promoting proliferation and osteolysis. Inhibiting HOXC10, paired with STAT3 blockade, disrupts compensatory IL-6/JAK/STAT3 signaling, leading to ROS accumulation, glutathione depletion, and ferroptosis, suppressing bone metastasis in models [37]. This tactic may extend to hepatic or cerebral metastases via similar defenses, meriting exploration [38, 39].

### KEAP1

KEAP1 mutations disrupt the KEAP1-NRF2 axis, causing persistent NRF2 activation, enhancing antioxidant defenses, and protecting against ferroptosis, supporting tumor survival. KEAP1-mutant NSCLC fosters an immune-cold microenvironment, reducing immunotherapy efficacy and increasing resistance to chemo, radiation, and targeted therapies [40]. In wild-type NRF2/KEAP1 lung squamous cell carcinoma (LUSC), ROS inducers trigger transient NRF2 activation, upregulating miR-126, which downregulates p85βPI3K and SET domain containing 5 (SETD5), disrupting survival signaling and inducing ferroptosis via pentose phosphate pathway overactivation. This approach, validated in cell lines and xenografts, suggests localized ROS delivery as a therapeutic option [41]. KEAP1-deficient cancers resist ferroptosis due to NRF2-driven FSP1 upregulation, converting CoQ to ubiquinol, neutralizing lipid radicals. Targeting the CoQ-FSP1 axis sensitizes cells to ferroptosis and radiation [42, 43]. NQO1 inhibition in KEAP1-deficient cancers disrupts antioxidant defenses, allowing lipid peroxide accumulation, inducing ferroptosis, and boosting CD8+ T cell-mediated immunity, enhancing PD-1 blockade [44]. Serine/threonine kinase 11 (STK11) /KEAP1 co-mutant LUAD resists ferroptosis via NRF2-driven antioxidant genes, STK11-mediated stearoyl-CoA desaturase 1 (SCD1) upregulation, and AKR1C activation, creating a multi-layered defense against oxidative stress [45].

### TP53

P53-mutant lung cancer cells resist ferroptosis, showing lower malondialdehyde, ferrous ions, and ROS compared to wild-type cells. Heparanase overexpression in TP53-mutant contexts enhances survival and vascular endothelial growth factor (VEGF)-driven angiogenesis, reinforcing ferroptosis insensitivity [46]. TP53 mutations disrupt ferroptosis sensitization. Wild-type TP53 suppresses FOXM1 via proliferator-activated receptor gamma coactivator 1-alpha (PGC-1α), but R248W mutations allow FOXM1 and myocyte enhancer factor 2C (MEF2C) upregulation, activating MAPK and enhancing ferroptosis resistance, preserving mitochondrial function, and supporting tumor progression [47].

### RIT1

Oncogenic mutations in RIT1, particularly hotspot mutations such as A77S, F82L, and M90I—result in increased protein stability and accumulation in lung adenocarcinoma cells, which in turn promote tumor growth by activating key oncogenic pathways like RAS/MAPK and PI3K/AKT. Intriguingly, these mutations also lead to a dysregulation of redox homeostasis by altering the expression of NRF2 downstream target genes, a disruption that renders the cancer cells more vulnerable to ferroptosis. Experimental studies have demonstrated that when these cells are treated with ferroptosis inducers such as RSL3, the elevated RIT1 levels significantly enhance ferroptotic cell death, suggesting that the oncogenic alterations in RIT1 not only drive tumor progression but also create a potential therapeutic window for targeting lung adenocarcinoma through ferroptosis induction [48].

### PTEN

Overexpression of ELF3 in the lung epithelium, when combined with PTEN mutations, creates a potent oncogenic environment by both enhancing cell proliferation and inhibiting ferroptosis. Under normal conditions, elevated ELF3 levels trigger not only hyperplasia but also ferroptosis. However, in the context of PTEN deficiency, ELF3 drives the upregulation of SLC7A11, a critical ferroptosis inhibitor, which in turn prevents ferroptotic cell death. This dual effect allows for unchecked cell growth while evading a key tumor-suppressive mechanism, thereby facilitating the development and progression of lung cancer [49].

### BRAF

BRAFV600E lung adenocarcinoma cells experience a profound shift in their redox homeostasis when subjected to targeted MAPK pathway inhibition, which leads to a drug-tolerant persister (DTP) state characterized by heightened oxidative stress and lipid peroxidation [50]. In this vulnerable state, the antioxidant enzyme GPX4 becomes critical for detoxifying harmful lipid hydroperoxides, and its inhibition triggers ferroptosis. This ferroptotic vulnerability is evidenced by increased levels of peroxidized lipids and intracellular iron, making early relapse tumors more susceptible to ferroptosis inducers such as RSL3. Ultimately, targeting GPX4 and inducing ferroptosis in these cells holds promise for delaying or overcoming drug resistance and reducing tumor progression in BRAFV600E lung adenocarcinoma [51].

### LKB1

STK11 (LKB1) mutations suppress ferroptosis in lung adenocarcinoma by reprogramming cellular lipid metabolism, specifically by promoting the synthesis of monounsaturated fatty acids (MUFAs) [45]. When STK11 is mutated or its expression is downregulated, there is an observed increase in the expression of key ferroptosis-inhibitory proteins, such as SLC7A11 and SCD1. SCD1, a rate-limiting enzyme in MUFA synthesis, is upregulated, leading to an accumulation of MUFAs that stabilize cell membranes against lipid peroxidation, a critical trigger of ferroptosis. Consequently, the protective effect of MUFAs mitigates the oxidative stress induced by agents like Erastin, thereby reducing reactive oxygen species, lipid peroxidation markers, and apoptosis. This metabolic reprogramming ultimately enables lung adenocarcinoma cells harboring STK11 mutations to evade ferroptotic cell death, underscoring a potential target for therapeutic intervention [52].

### SOX2

Mutations in the stem cell factor SRY-box transcription factor 2 (SOX2) can confer ferroptosis resistance in lung cancer by disrupting its normal regulatory role on the cystine transporter SLC7A11. Under typical conditions, SOX2 binds to a conserved site on the SLC7A11 promoter to transcriptionally upregulate its expression, which in turn enhances cystine uptake and promotes GSH [53, 54]. However, when mutations occur in SOX2 or its binding site on the SLC7A11 promoter, this regulatory axis is impaired. For instance, alterations that diminish SOX2 binding reduce SLC7A11 expression, leading to lower intracellular cysteine and GSH levels, increased lipid peroxidation, and heightened sensitivity to ferroptosis. Additionally, oxidative modification of SOX2 at its unique Cys265 residue (induced by cysteine deprivation) further hampers its transcriptional activity, thereby exacerbating the loss of ferroptosis resistance. This interplay illustrates how mutations affecting SOX2 not only compromise its role in maintaining cancer stem-like properties but also sensitize lung cancer cells to ferroptotic stress, suggesting potential therapeutic avenues to target this axis in treatment-resistant tumors [55].

## Hematological malignancies

**Fig. 3:**
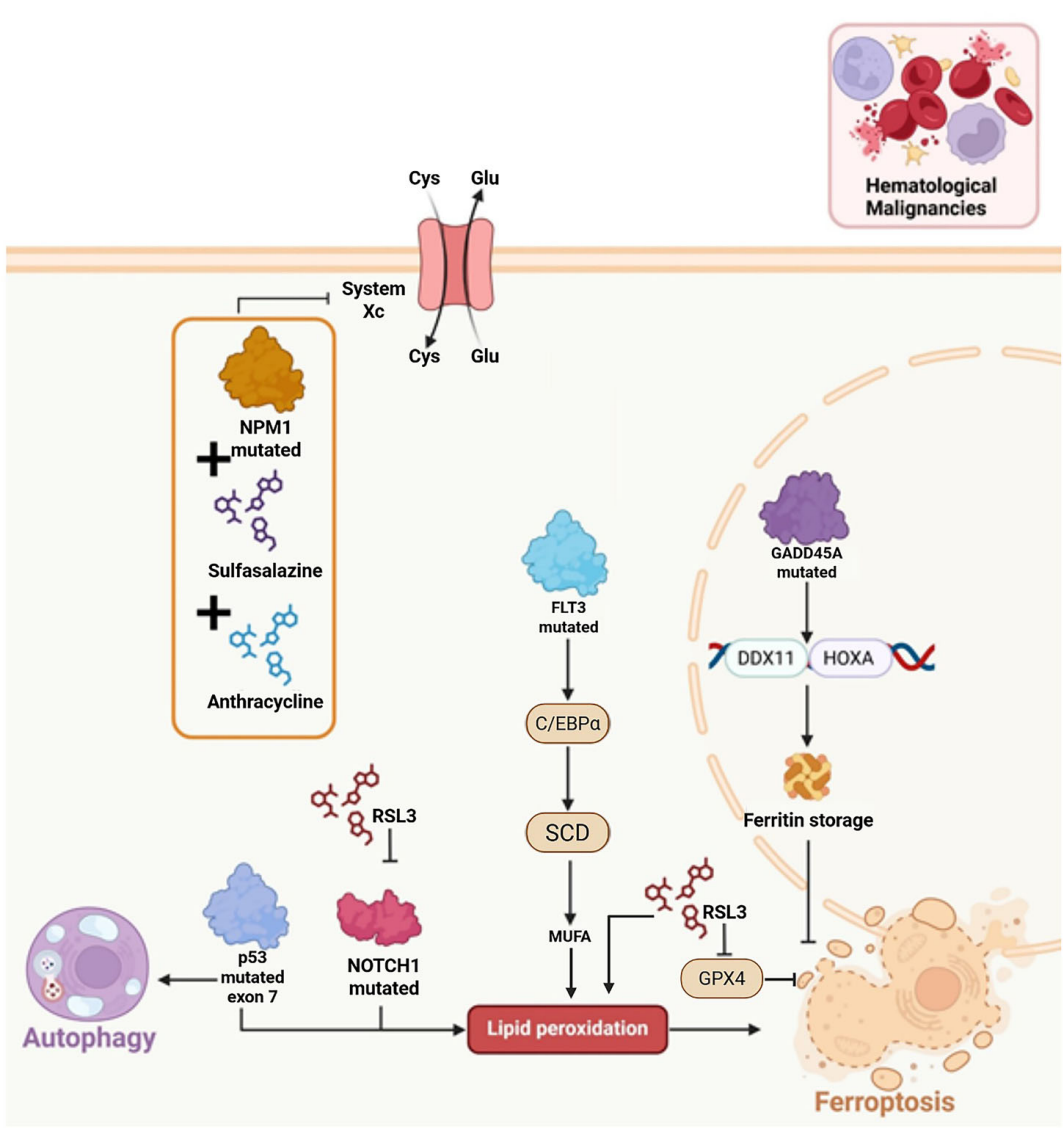
Overview of how recurrent gene mutations in hematological malignancies modulate ferroptosis. FLT3 variants promote MUFA production via CCAAT/enhancer-binding protein alpha (C/EBPα)- stearoyl-CoA desaturase (SCD), protecting against peroxidation; FLT3 blockers reverse this by axis suppression. GADD45A loss elevates FTH1 to trap iron and curb ROS, fostering resistance. NOTCH1 alterations (e.g., T-ALL) lower GPX4, heightening RSL3 sensitivity. TP53 variants unevenly induce ferroptosis or ferritinophagy (via NCOA4), with inactivating forms sensitizing cells. NPM1-mutant AML relies on xCT-mediated cystine influx, vulnerable to erastin blockade. These insights position ferroptosis modulation as a precision strategy for blood cancers.

Beyond mutational heterogeneity, hematological malignancies exhibit distinct, cell–type–specific vulnerabilities to ferroptosis. Normal immune cells, particularly T and B lymphocytes, are inherently prone to ferroptotic stress because of their abundant polyunsaturated lipids [56]. This intrinsic susceptibility likely explains patterns observed in genome-wide CRISPR screens, where B-cell acute lymphoblastic leukemia (B-ALL) cells show a marked dependence on ferroptosis-related pathways [57, 58]. Environmental context can further shape this sensitivity; for instance, central nervous system (CNS) involvement has been shown to influence ferroptosis responses. In addition, specific mutations, such as those in CREBBP, can modulate ferroptosis sensitivity within particular hematopoietic lineages [59–61]. Recognizing these broader determinants sets the stage for the following discussion of recurrent mutations, including FLT3, TP53, NPM1, Growth Arrest And DNA Damage Inducible Alpha (GADD45A), and Neurogenic locus notch homolog protein 1 (NOTCH1), that govern ferroptosis regulation in acute myeloid leukemia (AML), B-cell lymphoma, and T-lymphoblastic leukemia (T-ALL) [62]. Recurrent gene mutations in AML have significant clinical implications that enhance both risk stratification and treatment personalization. These mutations, such as FLT3-ITD, TP53, NPM1, and CEBPA, among others, not only influence the prognosis of patients, with certain mutations correlating with high relapse rates and poorer outcomes, but also serve as critical biomarkers for guiding therapeutic decisions (Fig. 3) [63].

### FLT3

FLT3-mutant leukemia exhibits a complex ferroptosis response driven by altered lipid metabolism. Constitutive FLT3 signaling enhances C/EBPα expression, upregulating lipogenic enzymes like fatty acid synthase (FASN), stearoyl-CoA desaturase (SCD), and fatty acid desaturase 2 (FADS2). These promote de novo fatty acid synthesis, maintaining high protective MUFAs in cell membranes. FLT3 inhibition reduces C/EBPα and these enzymes, decreasing MUFAs while increasing polyunsaturated fatty acids (PUFAs), shifting the PUFA/MUFA ratio. This makes membranes prone to lipid peroxidation, a key ferroptosis trigger. Combining FLT3 inhibitors with ferroptosis inducers like RSL3 or APR-246 (glutathione-depleting) heightens oxidative stress, synergistically killing leukemic cells in vitro and in patient-derived xenografts. This highlights FLT3-mutant leukemia’s ferroptosis vulnerability, offering a strategy to overcome resistance and target residual disease [64].

### GADD45A

GADD45A deletion increases replication stress and impairs DNA repair, leading to mutations in genes critical for genomic stability (e.g., DDX11, HOXA clusters). These trigger aberrant antioxidant defenses, notably upregulating FTH1, which sequesters iron and lowers ROS. Enhanced FTH1 reduces iron-driven ROS accumulation, mitigating oxidative damage that initiates ferroptosis. Thus, GADD45A loss destabilizes the genome and reprograms metabolism to resist ferroptotic death, fostering aggressive, therapy-resistant leukemia [65].

### NOTCH1

In T-ALL, prevalent NOTCH1-activating mutations drive oncogenic signaling and intersect with ferroptosis regulation. Ferroptosis inducers like erastin and RSL3 suppress NOTCH1 expression, reducing its active form and downstream HES1 in NOTCH1-mutant T-ALL cell lines. This dual effect, disrupting redox balance and promoting lipid peroxidation while inhibiting NOTCH1 signaling, makes cells more ferroptosis-susceptible. Notably, direct GPX4 knockdown does not affect NOTCH1, suggesting an indirect effect of ferroptosis induction [66].

### TP53

In diffuse large B-cell lymphoma (DLBCL), TP53 mutation type dictates ferroptosis response. Wild-type TP53 or non-exon 7 mutations lead to APR-246-induced ferroptosis, marked by increased ROS and lipid peroxidation, reversible by deferoxamine or ferrostatin-1. Exon 7 missense mutations trigger both ferroptosis and TP53-dependent ferritinophagy, enhancing anti-tumor effects through combined ferroptosis and ferritin degradation [67].

### NPM1

NPM1-mutated AML (e.g., OCI-AML3, IMS-M2) shows heightened xCT inhibition sensitivity despite similar SLC7A11 transcript levels, with greater cysteine pathway gene expression and cystine dependency. Sulfasalazine (SSZ) with daunorubicin synergistically boosts anti-leukemic activity. Ex vivo tests confirm low-dose SSZ with daunorubicin-cytarabine enhances cytotoxicity against AML cells and stem cells. In xenografts, this combination reduces leukemic burden and extends survival, highlighting cystine targeting’s potential [68].

## Liver cancer

**Fig. 4:**
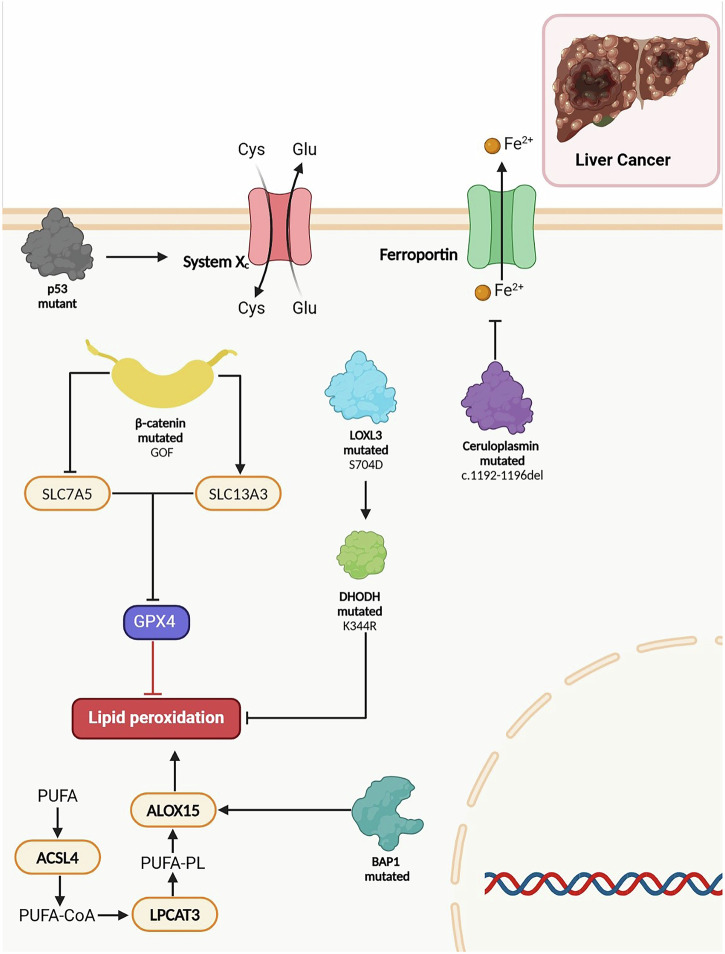
Ferroptosis regulation and mutational landscape in HCC. Mutations in TP53 lead to dysregulation of the cystine/glutamate antiporter (System Xc⁻), impacting glutathione homeostasis. Gain-of-function mutations in β-catenin (CTNNB1) upregulate SLC13A3, altering metabolic fluxes and impairing antioxidant defenses. Mutations in LOXL3 (S704D) and DHODH (K344R) stabilize DHODH, reducing lipid peroxidation and ferroptosis resistance. BAP1 mutations downregulate ferroptosis-related genes, contributing to tumor survival. The loss-of-function mutation in ceruloplasmin (c.1192-1196del) disrupts iron homeostasis, increasing intracellular Fe²⁺ levels. GPX4, a key inhibitor of lipid peroxidation, is functionally suppressed in HCC, rendering cells susceptible to ferroptotic cell death. These findings highlight the complex interplay between ferroptosis regulation and oncogenic mutations, emphasizing potential therapeutic targets in HCC treatment.

Hepatocellular carcinoma (HCC) exhibits a complex mutational landscape, characterized by frequent alterations in critical genes that drive tumorigenesis. Recurrent TERT promoter mutations activate telomerase, promoting unlimited cell proliferation [69]. TP53 mutations, often associated with poor prognosis, compromise genomic integrity. Mutations in the WNT pathway, particularly in CTNNB1 and its regulator AXIN1, trigger aberrant signaling and cellular growth. Additionally, loss-of-function mutations in chromatin remodelers ARID1A and ARID2, alongside alterations in oxidative stress regulators NFE2L2 and KEAP1, further contribute to HCC progression. These diverse genetic changes underscore the necessity for personalized therapeutic strategies to address HCC’s complexity (Fig. 4) [70].

### β-catenin

Gain-of-function mutations in CTNNB1 hyperactivate β-catenin signaling, increasing expression of downstream effector SLC13A3. This enhances uptake of tricarboxylic acid (TCA) cycle intermediates and GSH but disrupts amino acid homeostasis, particularly by reducing leucine levels through c-MYC-mediated downregulation of the leucine transporter SLC7A5. The resulting GSH depletion impairs the GSH-GPX4 axis, which normally detoxifies lipid peroxides, thereby increasing vulnerability to lipid peroxidation and promoting ferroptosis. This mechanism presents a promising therapeutic target for β-catenin-driven HCC [71].

### LOXL3

In HCC, Lysyl Oxidase Like 3 (LOXL3) phosphorylation at serine 704 enhances its lysyl-oxidase activity, stabilizing dihydroorotate dehydrogenase (DHODH) by preventing ubiquitin-mediated degradation at lysine 344 [72]. The phospho-mimetic LOXL3 mutation (S704D) reduces lipid peroxidation, suppressing ferroptosis and fostering chemoresistance. Conversely, the dephosphorylation mimic (S704A) impairs LOXL3 activity, leading to DHODH degradation, heightened lipid peroxidation, and increased ferroptosis. A DHODH mutation at lysine 344 (K344R) similarly prevents ubiquitination, reinforcing ferroptosis resistance and influencing chemotherapy outcomes [73].

### TP53

Wild-type p53 promotes ferroptosis by repressing SLC7A11, a cystine/glutamate antiporter component critical for glutathione synthesis. TP53 mutations abolish this repression, sustaining SLC7A11 expression, enhancing cystine uptake, and elevating glutathione levels. This reduces lipid peroxidation, conferring ferroptosis resistance and contributing to chemoresistance in HCC, thus impacting therapeutic efficacy [74]. In HCC, CAPG acts as an upstream regulator, promoting p53 degradation through WDR74-mediated enhancement of MDM2 binding, which disrupts p53 stability and elevates SLC7A11 to suppress ferroptosis. This pathway drives tumor growth and sorafenib resistance, as CAPG knockdown restores p53 function, boosts lipid peroxidation, and sensitizes cells to therapy, highlighting a targetable axis for improved outcomes [75].

### BAP1

Somatic BAP1 mutations in HCC, including splicing, nonsense, missense, and frameshift alterations, reduce BAP1 expression, lowering levels of ferroptosis-related genes, Arachidonate 15-Lipoxygenase (ALOX15), FDFT1, NCOA4, and DPP4. This diminishes ferroptosis capacity. High BAP1 expression, conversely, enhances ferroptosis signaling but correlates with an immunosuppressive tumor microenvironment and poorer clinical outcomes, suggesting BAP1’s potential as a prognostic biomarker for ferroptosis-based or immune therapies [76].

### Ceruloplasmin

Knockdown of ceruloplasmin (CP) in HCC cells, HepG2 and Hep3B, increases intracellular ferrous iron, lipid ROS, and malondialdehyde (MDA), while reducing glutathione, promoting ferroptotic cell death. Overexpression of wild-type CP maintains iron homeostasis, suppressing ferroptosis. A frameshift mutation (c.1192-1196del, p.Leu398Serfs) impairs CP’s iron export function, sustaining ferroptosis. CP’s protective role relies on ferroportin, while blocking iron import via TFRC mitigates ferroptosis [77].

## Colorectal cancer

Mutations are a major driver of therapeutic resistance in colorectal cancer (CRC) by disrupting key signaling pathways and interfering with drug-target interactions. For instance, activating mutations in genes such as KRAS, NRAS, and BRAF lead to continuous stimulation of downstream pathways (e.g., RAS/RAF/MEK/ERK), which makes therapies that target upstream receptors like EGFR ineffective. Additionally, mutations affecting genes involved in the PI3K/AKT pathway or drug metabolism can alter drug activation, increase drug efflux, or impair apoptotic responses, further contributing to resistance [78].

### KRAS

**Fig. 5:**
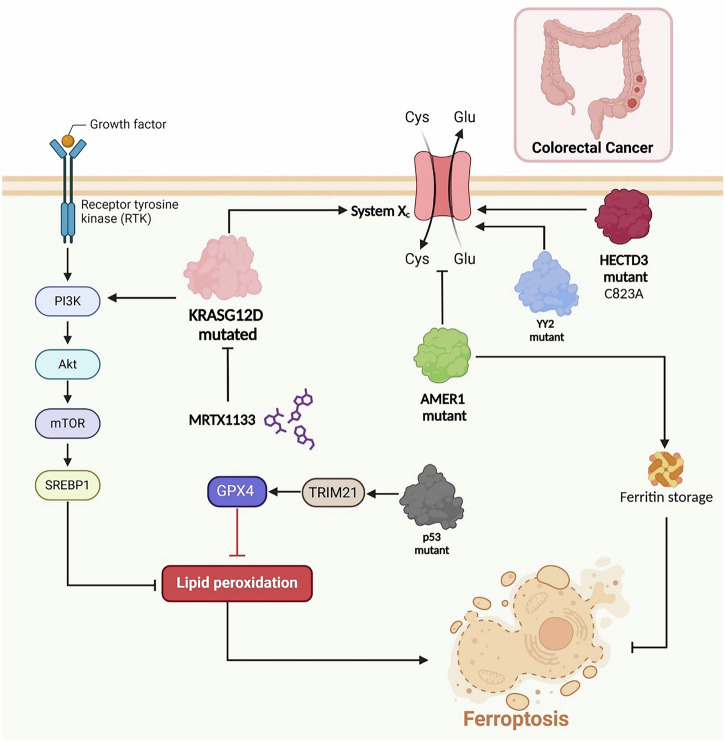
Ferroptosis regulation in colorectal cancer and oncogenic mutations. KRASG12D mutations enhance cystine uptake via System Xc⁻, increasing glutathione synthesis and reducing oxidative stress. However, treatment with the KRAS inhibitor MRTX1133 promotes ferroptosis by elevating lipid peroxidation. PI3K/AKT/mTOR signaling, activated by receptor tyrosine kinases, regulates SREBP1, a key player in lipid metabolism that further modulates ferroptosis resistance. Mutant p53 interacts with TRIM21, stabilizing GPX4 and preventing lipid peroxidation. Loss-of-function mutations in AMER1 impair iron metabolism and ferritin storage, reducing susceptibility to ferroptosis. Similarly, mutations in HECTD3 (C823A) and YY2 dysregulate SLC7A11, leading to ferroptosis resistance. These molecular alterations collectively contribute to ferroptosis evasion and tumor progression, highlighting potential therapeutic targets for CRC treatment.

Oncogenic KRAS mutations, exemplified by G12D, consistently upregulate the cystine importer SLC7A11 across diverse cancers, bolstering glutathione production and fortifying cellular antioxidant capacity. This mechanism, observed in colorectal, lung, and pancreatic models, supports tumor adaptation to oxidative challenges but yields variable impacts on steady-state ferroptosis [79]. In CRC, such alterations fail to markedly shift baseline ferroptosis susceptibility; rather, TP53 inactivation exerts a stronger influence by amplifying SLC7A11 activity, thereby shielding cells from iron-catalyzed lipid damage. Consequently, KRAS variants drive expansion via nutrient scavenging and metabolic shifts, yet their ferroptosis modulation in CRC remains secondary to p53 disruption [80]. Notably, KRAS^G12D blockade with MRTX1133 elicits ferroptosis through iron overload, ROS escalation, glutathione disequilibrium, and heightened lipid oxidation, eroding membrane stability to curb proliferation and spread. This approach disrupts KRAS/G12D-fueled pathways effectively [81]. Furthermore, in KRAS-altered CRC, TYM-3-98-mediated PI3Kδ blockade curbs AKT/mTOR signaling, diminishing Sterol regulatory element-binding protein 1 (SREBP1)-driven lipid synthesis and fostering ferroptosis via lipid oxidation surge, iron buildup, and glutathione exhaustion. Experimental data affirm that KRAS changes engender ferroptosis proneness, counteracted by SREBP1 excess; its silencing amplifies TYM-3-98’s ferroptotic potency (Fig. 5) [82].

### TP53

In TP53-mutant colon cancers, MDM4 overexpression correlates with poor prognosis and promotes tumor growth and chemoresistance by inhibiting ferroptosis. In cell lines like HT29 and SW480, MDM4 stabilizes GPX4 protein, without altering its mRNA, by upregulating the E3 ubiquitin ligase Tripartite Motif Containing 21 (TRIM21). TRIM21 shifts GPX4 ubiquitination from degradative K48-linked to non-degradative K63-linked forms, preserving GPX4 to detoxify lipid peroxides and block ferroptosis. Inhibiting MDM4 with NSC146109, combined with RSL3, restores ferroptosis sensitivity, reducing tumor growth in xenograft models. Targeting the MDM4-TRIM21/GPX4 axis thus offers a promising approach for TP53-mutant CRC [83].

### AMER1

APC Membrane Recruitment Protein 1 (AMER1) mutations in CRC disrupt its function, reducing ubiquitination and degradation of SLC7A11 and ferritin light chain (FTL). Elevated SLC7A11 increases cystine uptake and GSH synthesis, while FTL accumulation limits free iron for ROS generation. This reduces lipid peroxidation, conferring ferroptosis resistance and supporting metastatic survival under oxidative stress [84].

### HECTD3

HECTD3, an E3 ubiquitin ligase, suppresses CRC by promoting SLC7A11 ubiquitination and degradation, limiting cystine uptake, and enabling ferroptosis. When HECTD3 is downregulated or harbors a C823A mutation, its ubiquitinase activity falters, stabilizing SLC7A11, boosting GSH, and suppressing lipid peroxidation. This inhibits ferroptosis, accelerating tumor progression in vitro and in vivo [85].

### YY2

Mutations in the zinc-finger domains of YY2 critically impair its ability to bind to and repress the SLC7A11 promoter [86]. In colorectal cancer patients, this loss of function results in increased SLC7A11 expression, leading to enhanced cystine uptake and glutathione synthesis. Consequently, tumor cells acquire a stronger antioxidant capacity that protects them from lipid peroxidation and ferroptotic cell death. This resistance to ferroptosis not only promotes tumor cell survival but also contributes to more aggressive tumor progression and poorer clinical outcomes (Fig. 5) [87].

## Breast cancer

**Fig. 6:**
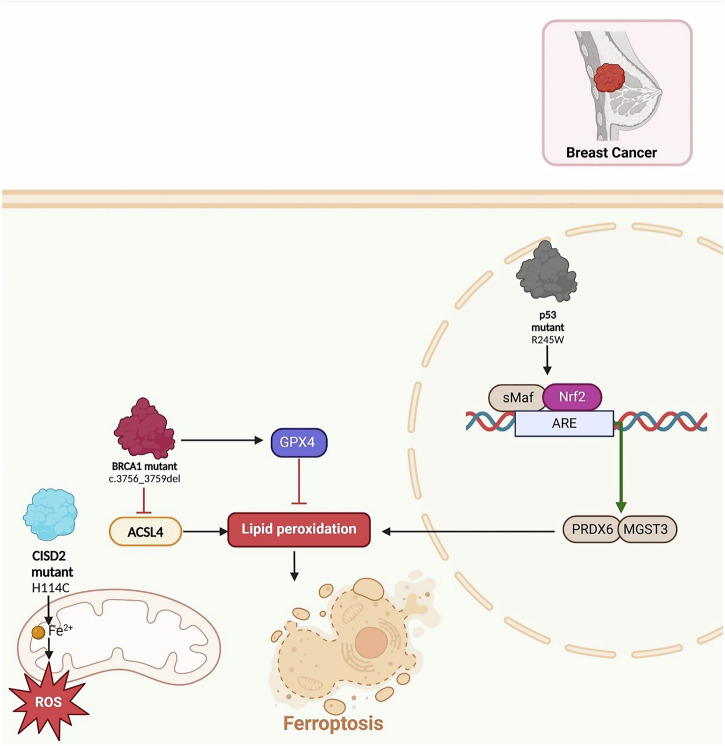
Ferroptosis regulation and genetic mutations in breast cancer. BRCA1 mutations (c.3756_3759del) impair the degradation of GPX4, a crucial inhibitor of lipid peroxidation, thereby conferring resistance to ferroptosis. CISD2 mutations (H114C) disrupt mitochondrial iron homeostasis, leading to ROS accumulation and increased ferroptosis sensitivity. Mutant p53 (R245W) activates the Nrf2-ARE pathway, upregulating antioxidant enzymes peroxiredoxin-6 (PRDX6) and microsomal glutathione S-transferase 3 (MGST3), which suppress lipid peroxidation and ferroptotic cell death. These molecular events underscore the complex interplay between tumor genetics and ferroptosis regulation, offering insights into potential therapeutic vulnerabilities in breast cancer treatment.

Genetic alterations significantly influence breast cancer by fueling tumor initiation and advancement, while also shaping therapeutic responses. Changes in pivotal genes can modify drug metabolism, cellular efflux mechanisms, and the modulation of signaling cascades [88]. For example, TP53 mutations or variants in transporters like ABCB1 may diminish chemotherapy effectiveness, whereas modifications in enzymes such as CYP2D6 could hinder the activation of hormone-based treatments. Such genomic variations account for diverse clinical results and foster resistance to drugs, emphasizing the importance of individualized approaches based on a patient’s specific genetic makeup (Fig. 6) [89].

### BRCA1

BRCA1 haploinsufficiency in breast cancer disrupts DNA double-strand break repair, increasing vulnerability to oxidative stress from the Fenton reaction, where excess iron generates ROS and lipid peroxidation [90]. Normally, lipid peroxide buildup triggers ferroptosis, but BRCA1 deficiency alters mitochondrial function and iron regulation, modifying ACSL4 and Cox-2 levels, increasing ferritin iron storage, and disrupting transferrin expression. These changes limit lethal lipid peroxidation, conferring ferroptosis resistance, allowing DNA damage propagation and chromosomal amplifications like c-Myc, fueling aggressive tumor growth [90]. BRCA1 also regulates ferroptosis by controlling GPX4 stability. In BRCA1-proficient cells, BRCA1’s RING domain induces K6-linked polyubiquitination of GPX4 at K47 and K58, promoting its degradation and enhancing lipid peroxidation for ferroptosis. In BRCA1-deficient cells or those with ligase-impaired mutations, elevated GPX4 levels reduce ferroptosis, diminishing PARP inhibitor efficacy. In models like UWB1.289 and A2780 cells, and BRCA1 c.3756_3759del organoids, GPX4 inhibition restores sensitivity to PARP therapy [91].

### TP53

Missense p53 mutations, like p53R172H and p53R245W, protect triple-negative breast tumors from ferroptosis. In mouse models, mutant p53 deletion caused tumor regression with lipid droplet accumulation and elevated 4-hydroxynonenal, indicating ferroptosis. Cells lacking mutant p53 were sensitive to inducers like RSL3, reversible by liproxstatin-1. Single-cell analysis showed mutant p53 activates NRF2, upregulating antioxidants MGST3 and PRDX6, mimicking GPX4’s role in detoxifying lipid peroxides. In human cell lines, silencing mutant TP53 reduced these enzymes, increasing ferroptosis sensitivity, with high NRF2 antioxidant expression linked to poorer survival [92].

### CISD2

CDGSH iron-sulfur domain 2 (CISD2) mutations, such as H114C, disrupt mitochondrial iron homeostasis in breast cancer, causing rapid labile iron accumulation and ROS surge. This upregulates TXNIP, lowers GPX4, increases transferrin receptor, and elevates lipid peroxidation, promoting ferroptosis. Loss of CISD2 function thus heightens susceptibility to iron-dependent cell death [93].

## Glioma

**Fig. 7:**
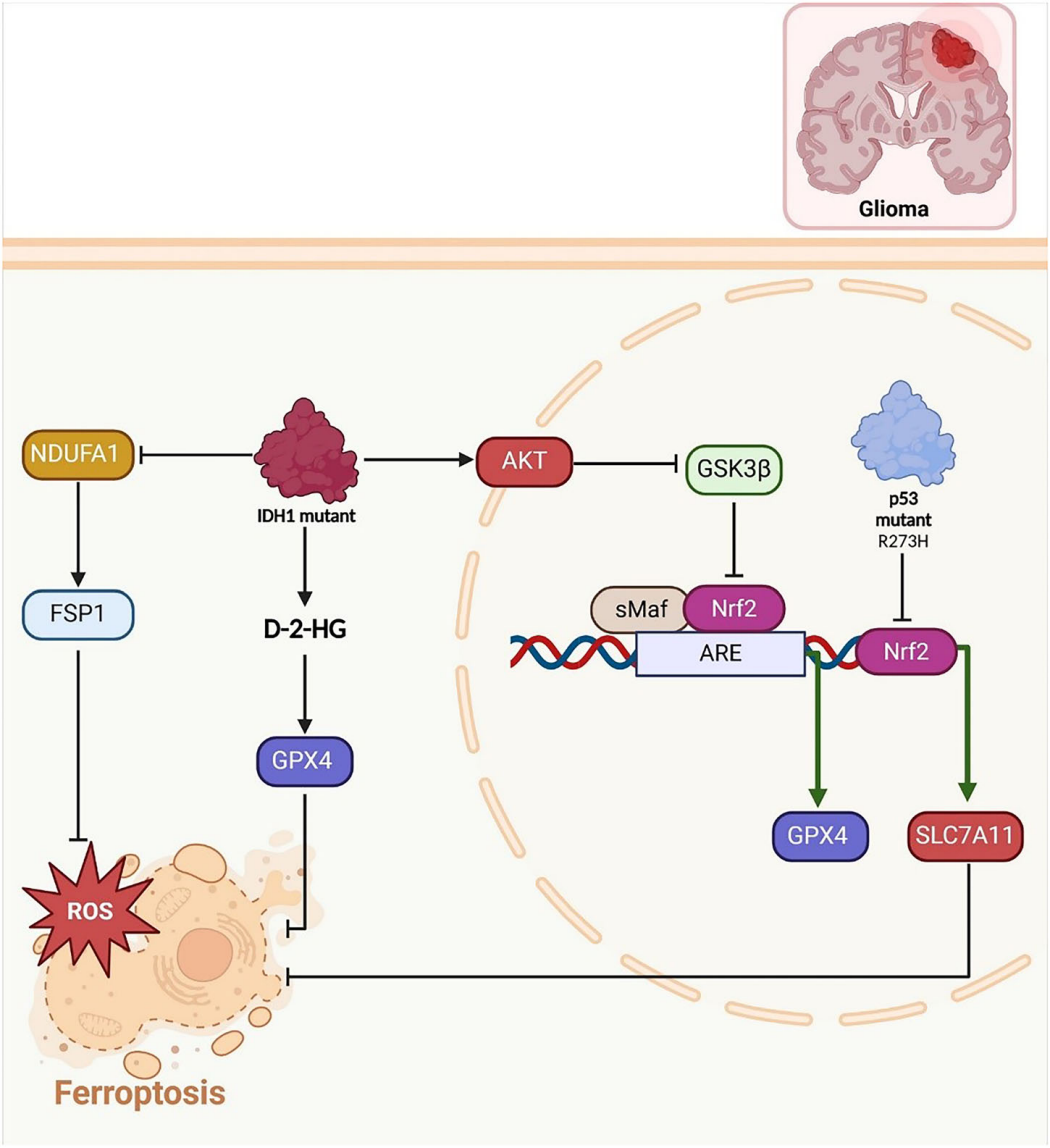
Ferroptosis regulation and oncogenic mutations in glioma. IDH1 mutations drive the production of D-2-HG, which suppresses GPX4, leading to lipid peroxidation and ferroptotic susceptibility. However, mutant IDH1 also activates the AKT pathway, inhibiting GSK3β and stabilizing Nrf2, which enhances antioxidant defenses and ferroptosis resistance. p53 mutations (R273H) further potentiate ferroptosis evasion by increasing Nrf2-dependent expression of SLC7A11 and GPX4, thereby mitigating oxidative stress. Additionally, downregulation of NDUFA1 disrupts mitochondrial function, contributing to ferroptotic vulnerability. These findings highlight key molecular interactions that shape ferroptosis sensitivity in glioma and offer potential therapeutic targets for treatment.

IDH1 mutations in gliomas drive metabolic and epigenetic reprogramming, producing D-2-hydroxyglutarate (D-2-HG), which depletes α-ketoglutarate, disrupts redox balance, and alters energy and glutamine metabolism. This induces hypermethylation (G-CIMP), affecting gene expression and differentiation. Clinically, IDH mutations are linked to better prognosis, serving as diagnostic markers and targets for tailored therapies (Fig. 7) [94].

### IDH1

These mutations sensitize cells to ferroptosis by converting α-ketoglutarate to D-2-HG, reducing GPX4 levels, a key lipid ROS scavenger, and depleting glutathione, leading to lipid peroxide accumulation and ferroptotic cell death. Inhibiting mutant IDH1 or overexpressing D2HGDH reduces D-2-HG, attenuating ferroptosis sensitivity, highlighting exploitable tumor vulnerabilities [95]. In IDH-mutated gliomas, AKT enhances Nrf2-driven antioxidant defenses, protecting against ferroptosis. D-2-HG activates AKT, which phosphorylates GSK3β, stabilizing Nrf2 by blocking its degradation. Nrf2 then upregulates genes for glutathione synthesis and ROS scavenging, minimizing lipid peroxidation and promoting survival [96]. Additionally, IDH1-R132H causes NADH:ubiquinone oxidoreductase subunit A1 (NDUFA1) promoter hypermethylation, reducing NDUFA1 expression and disrupting its FSP1 interaction, impairing mitochondrial ROS clearance. This increases ROS, lipid peroxidation, and cisplatin-induced ferroptosis in renal cells, offering insights for personalized therapies to mitigate nephrotoxicity [97].

### TP53

The study by Yuan et al. demonstrated that p62 plays a dual role in regulating ferroptosis in glioblastoma depending on the p53 status. In GBM cells harboring p53 mutations (such as R273H or R213Q), p62 overexpression leads to reduced SLC7A11 expression and increased lipid peroxidation, factors that promote ferroptotic cell death. This effect is mediated by an enhanced association between mutant p53 and NRF2, which suppresses NRF2’s antioxidant function. Conversely, in p53 wild-type GBM cells, p62 activates the conventional p62–NRF2 signaling pathway, resulting in elevated SLC7A11 expression and a protective, anti-ferroptotic effect. These findings suggest that p53 mutation status is a critical determinant for the therapeutic response to p62-mediated, ferroptosis-targeted strategies in glioblastoma [98].

## Renal cell carcinoma

**Fig. 8:**
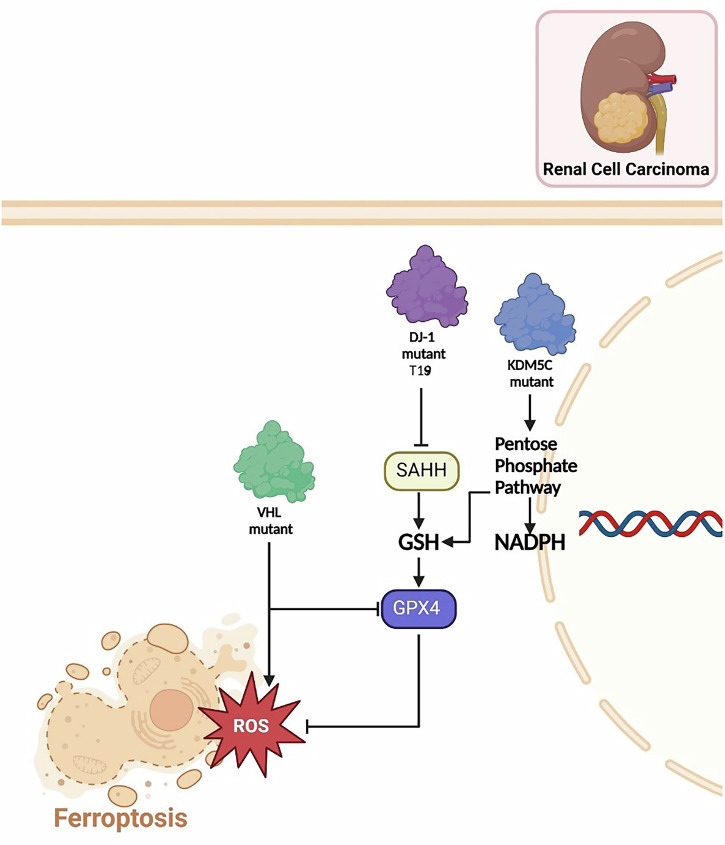
Ferroptosis regulation and genetic mutations in renal cell carcinoma. DJ-1 mutations (T19) impair the SAHH-mediated production of GSH, reducing antioxidant defenses and promoting ferroptotic cell death. Loss of VHL function leads to increased ROS accumulation and downregulation of GPX4, further sensitizing cells to ferroptosis. Meanwhile, KDM5C mutations enhance the pentose phosphate pathway (PPP), boosting NADPH and GSH levels, thereby conferring ferroptosis resistance. These genetic alterations collectively regulate the oxidative stress balance in RCC, influencing tumor survival and therapeutic vulnerabilities.

Genetic mutations drive renal cell carcinoma (RCC), shaping tumor growth, spread, and therapy responses. In clear cell RCC (ccRCC), von Hippel-Lindau (VHL) loss boosts hypoxia-inducible factor (HIF) and VEGF, spurring angiogenesis. PBRM1, BAP1, and SETD2 mutations refine prognosis and targeted therapy or immunotherapy success. Papillary RCC’s MET alterations enable MET inhibitors, while sarcomatoid RCC’s TP53 and NF2 changes signal aggression but immunotherapy potential. These genomic advances overhaul RCC care, yet biomarker discovery for precision medicine endures (Fig. 8) [99].

### DJ-1

Mutations in DJ-1, such as the T19A mutation, disrupt O-GlcNAcylation at the T19 residue, destabilizing its homodimeric structure and impairing antioxidant functions. Normally, O-GlcNAcylation supports homocysteine synthesis via the transsulfuration pathway. The T19A mutation enhances interactions between S-adenosyl homocysteine hydrolase (SAHH) and its inhibitor AHCYL1, reducing SAHH activity and GSH production. This GSH depletion elevates lipid ROS, triggering ferroptosis and underscoring DJ-1’s role in oxidative defense [100].

### VHL

In ccRCC, VHL mutations stabilize HIF-2α, activating the HIF-HILPDA axis that selectively enriches polyunsaturated fatty acids in lipid droplets and phospholipids, fostering GPX4 dependency and ferroptosis susceptibility. Concurrently, loss of VHL upregulates lipocalin-2 (LCN-2), elevating lipid ROS and disrupting iron homeostasis while suppressing GPX4. This synergy heightens oxidative damage, igniting JNK-mediated inflammation and reshaping the tumor microenvironment [101, 102].

### KDM5C

Lysine-specific demethylase 5C (KDM5C) frameshift mutations in ccRCC enhance ferroptosis resistance by increasing glycogen accumulation, which fuels the pentose phosphate pathway (PPP). This boosts NADPH and GSH production, protecting cells from ROS and ferroptosis. Restoring wild-type KDM5C reduces glycogen and PPP flux, sensitizing cells to ferroptosis, while the H514A mutant, lacking demethylase activity, fails to do so, highlighting KDM5C’s role in tumorigenesis [103].

## Other cancers

Beyond the primary malignancies discussed, a spectrum of lesser-explored cancers reveals nuanced genetic influences on ferroptosis dynamics, underscoring the pathway’s broad therapeutic relevance. In pancreatic and cholangiocarcinomas, KRAS variants heighten redox instability, fostering GPX4 dependency and vulnerability to inducers amid metabolic rewiring [104, 105]. Thyroid tumors with BRAFV600E mutations bolster antioxidant shields via ARSI-STAT3 signaling, while prostate SPOP alterations sustain glutathione flux through JMJD6-ATF4, evading peroxidation [106]. Cervical KDM4A disruptions impair SUMOylation-driven defenses, sensitizing cells; gastric E-cadherin loss unleashes Hippo-YAP vulnerability; and melanomas harboring mutp53(E285K) paradoxically amplify GPX4 inhibitor potency despite chemoresistance [107]. Ovarian p53 mutants blunt repression of SLC7A11, promoting evasion, whereas Wilms tumor DROSHA changes elevate ACSL4 for enhanced susceptibility, and meningioma NF2 deficiencies yield Erastin-responsive states tempered by E-cadherin [108, 109]. These patterns highlight mutation-tailored ferroptosis exploitation; detailed mechanisms and strategies appear in Table 1.

## Targeting mutations to enhance ferroptosis therapy

**Fig. 9:**
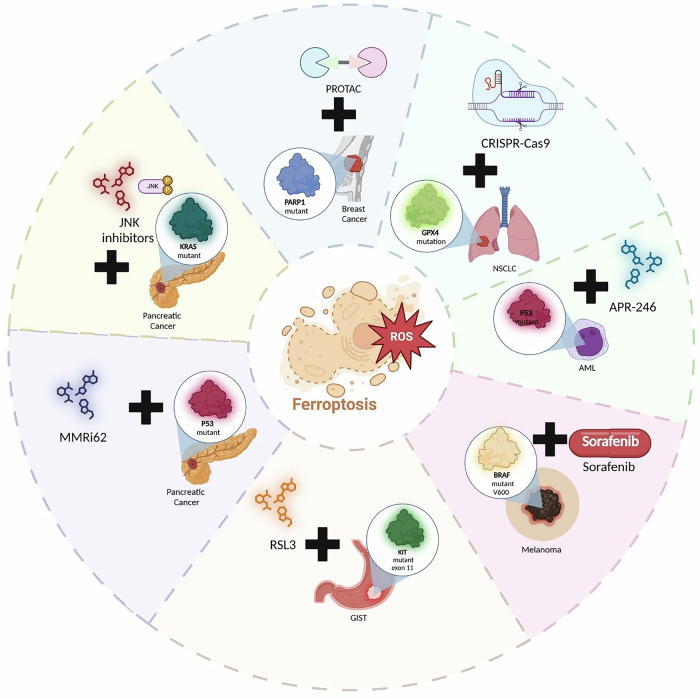
Targeting oncogenic mutations using synthetic drugs and strategies to enhance ferroptosis-based cancer therapy.

In the evolving landscape of precision oncology, targeting genetic mutations offers a smarter way to combat cancer, delivering treatments that zero in on tumor-specific flaws while sparing healthy cells. This contrasts sharply with the broad strokes of traditional chemotherapy, which often bring harsh side effects. Breakthroughs in next-generation sequencing now allow us to map out actionable mutations in real time, paving the way for tailored therapies that pack a bigger punch. We’ve seen this in action with drugs like imatinib revolutionizing chronic myelogenous leukemia and EGFR inhibitors transforming non-small cell lung cancer care (Reviewed by [6]). By tackling resistance head-on and blending these therapies with others, we’re unlocking even greater potential. Enter ferroptosis, an iron-fueled cell death process powered by lipid peroxidation, that shines when matched with mutation-targeted strategies, turning cancer’s own weaknesses against it. (Table 2) (Fig. 9).

**Table 2:** Therapeutic strategies for inducing ferroptosis in cancers with specific mutations.

Drug/strategy name	Cancer	Cell line	Model	Mutation	Effect on ferroptosis	Ref
**NN3**	Triple-negative breast cancer	MDA-MB-231	In vitro transfection model	R591C/848delY/T910A	Induces robust ferroptosis by degrading mutant PARP1, which downregulates the SLC7A11/GPX4 axis, leading to enhanced lipid peroxidation and cell death in p53-positive cells.	[110]
**RSL-3** **+** **JNK Inhibitors (JNK-IN-8/SP600125)**	Fibrosarcoma (as a model for cancer)	HT-1080	In vitro cell culture model	NRAS mutation	Co-treatment with JNK inhibitors amplifies RSL-3-induced ferroptosis in HT-1080 cells, significantly reducing cell viability through enhanced lipid peroxidation while leaving ROS levels unchanged.	[111]
**RSL-3** **+** **SP600125**	Pancreatic cancer	MIA PaCa-2	In vitro cell culture model	KRAS mutation	In MIA PaCa-2 cells, combining RSL-3 with a JNK inhibitor further decreases cell viability by deepening ferroptosis, an effect that is reversible with ferroptosis inhibitors.	
**MMRi62**	Pancreatic ductal adenocarcinoma (PDAC)	Panc1, BxPc3, etc.	In vitro cell culture and orthotopic xenograft mouse models	KRAS mutation (e.g., KRAS^G12D in Panc1) and mutant TP53 (e.g., p53^R273H in Panc1, p53^Y220C in BxPc3)	Induces ferroptosis by promoting lysosomal degradation of ferritin heavy chain (FTH1) and proteasomal degradation of mutant p53, leading to increased ROS production, enhanced autophagy, disrupted iron homeostasis, and ultimately, cell death and inhibition of metastasis.	[112]
**RSL3**	Gastrointestinal stromal tumour (GIST)	GIST-T1	In vitro imatinib-derived persister cells and subcutaneous xenograft model	KIT exon 11 heterozygous deletion (EGFR)	Induces ferroptosis in persister cells by exploiting downregulated GPX4 and depleted GSH levels, leading to iron-dependent, caspase-independent lipid peroxidation. The effect is confirmed by rescue with deferoxamine and ferrostatin-1, and it suppresses tumor regrowth following imatinib treatment.	[113]
**CRISPR-Cas9 mediated GPX4 knockout**	NSCLC	NSCLC cell lines	Gene editing model	Loss-of-function mutation in GPX4	Induces ferroptosis through loss of GPX4’s antioxidant defense, increasing ROS and lipid peroxidation	[114]
**APR-246**	AML	MOLM14, OCI-AML2, etc.	In vitro & in vivo	Mutant p53	Reactivates mutant p53 via conversion to MQ, depletes intracellular glutathione, increases ROS and lipid peroxidation, and thereby induces early ferroptotic cell death	[115]
**Combination: Vemurafenib + Sorafenib**	Melanoma	A375/Vem, SK-Mel-28/Vem	In vitro	BRAF mutations (e.g., BRAFV600E) driving resistance	Synergistically increases ROS production, raises iron and malondialdehyde (MDA) levels, and depletes GSH, thereby promoting ferroptosis. This cell death is blocked by the ferroptosis inhibitor Ferrostatin-1.	[139]
**Erianin**	Colorectal cancer	LoVo, HCT116, DLD-1, HCT-8	In vitro (cell viability, migration/invasion assays) and In vivo (xenograft and liver metastasis models using HCT116 cells)	KRAS^G13D	Induces autophagy-dependent ferroptosis by increasing Fe²⁺, ROS, and lipid peroxidation while modulating ferroptosis-related proteins (e.g., downregulating GPX4 and FTH1)	[117]
**Compound 16**	Thyroid cancer	MDA-T32	In vitro	BRAFV600E	Inhibits GPX4 expression, thereby inducing ferroptosis	[140]
**β-Elemene + Erlotinib**	NSCLC	H1975, H1650	In vitro and In vivo (xenograft)	EGFR-mutant (primary resistance to EGFR-TKIs)	Combination treatment enhances erlotinib sensitivity by upregulating lncRNA H19, which in turn promotes ferroptosis via increased ROS, lipid peroxidation, GSH depletion, and downregulation of GPX4 and other ferroptosis defense proteins.	[118]
**β-Elemene + cetuximab**	Colorectal cancer	HCT116, Lovo	In vitro and in vivo (orthotopic xenograft)	KRAS mutant	Induces ferroptosis by elevating ROS and lipid peroxidation, depleting GSH, and downregulating ferroptosis defense proteins (e.g., GPX4, SLC7A11, FTH1), thereby sensitizing cells to treatment and inhibiting EMT.	[119]
**Bromelain**	Colorectal cancer	HCT116, DLD-1	In vitro cell cultures and KRAS mutant mouse model with DSS-induced carcinogenesis	KRAS mutations (G12D, G13D)	Induces ferroptosis by upregulating ACSL-4, which increases lipid ROS accumulation and lipid peroxidation, thereby promoting ferroptotic cell death in KRAS mutant cells.	[120]
**Realgar**	Non-small cell lung cancer (NSCLC)	H23 (KRAS mutant), A549, H460, H1650 (non-KRAS mutant)	In vitro cell cultures and mouse xenograft models	KRAS mutations (e.g., in H23 cells)	Induces ferroptosis by increasing intracellular iron (Fe²⁺), ROS, and MDA levels while depleting GSH. It downregulates ferroptosis defense proteins (GPX4 and SLC7A11) and upregulates ACSL4, mediated via modulation of the Ras/Raf/MAPK signaling pathway.	[121]
**Betulin + Gefitinib**	NSCLC	A549, H460	In vitro cell cultures and xenograft model	EGFR wild-type/KRAS-mutant	The combination treatment induces ferroptosis by significantly increasing intracellular reactive oxygen species (ROS) and malondialdehyde (MDA) levels while depleting GSH. It downregulates ferroptosis negative regulators such as GPX4, SLC7A11, and FTH1, and upregulates HO-1. Ferroptosis inhibitors can abrogate these effects, confirming that the cell death induced by the combination is mainly due to ferroptosis	
**Co8FeS8@Co1-xS Nanoenzymes**	Liver cancer	H22 (murine hepatoma cells)	In vitro cell culture and in vivo xenograft (KM mice)	KRAS mutation	Induces a synergistic apoptosis-ferroptosis cell death by depleting intracellular GSH, inactivating GPX4, and increasing ROS and lipid peroxidation through its peroxidase-like and glutathione oxidase activities.	[123]

### Syntehtic drugs

Synthetic drugs are at the forefront, cleverly exploiting mutations to ignite ferroptosis. For example, PROTACs dismantle mutant PARP1 in breast cancer, crippling DNA repair and antioxidant safeguards to spark cell death [110]. Pairing JNK inhibitors with inducers like RSL-3 ramps up lipid damage in NRAS/KRAS-driven tumors [111], while MMRi62 hits mutant p53 and iron storage proteins in pancreatic cancer to unleash oxidative chaos [112]. These innovations, from RSL3 in resistant gastrointestinal stromal tumors [113] to CRISPR tweaks and APR-246 in lung and blood cancers [114, 115], highlight how synthetic tools can tip the scales toward ferroptosis.

### Natural products

Nature steps in with equally potent allies, disrupting cancer’s redox balance to fuel ferroptosis. Berberine weakens key defenses in p53-intact lung cancers [116], and erianin latches onto mutant KRAS in colorectal tumors for an autophagy-boosted kill [117]. Compounds like β-elemene, bromelain, realgar, and betulin further sensitize EGFR/KRAS-mutant cells by hiking ROS and peroxidation [118–122]. These natural gems pair seamlessly with synthetics, broadening our arsenal. For a deeper dive into these strategies, see Table 2.

### Nanoparticles

Derived from metal-organic frameworks, Co8FeS8@Co1-xS nanoenzymes exhibit dual mimicry of peroxidase and glutathione oxidase enzymes, creating a self-sustaining cascade for cancer treatment. Tailored to combat apoptosis-resistant cells driven by KRAS mutations, they were tested in KRAS-altered H22 hepatocellular carcinoma lines. Their distinctive heterojunction accelerates electron flow, transforming scarce tumor hydrogen peroxide into deadly hydroxyl radicals (•OH). Concurrently, glutathione depletion by their oxidase function undermines cellular antioxidants, disabling GPX4 and promoting unchecked lipid peroxidation. This orchestrated assault fuses apoptosis, via Bcl-2 suppression and caspase activation, with ferroptosis, marked by iron-fueled ROS surges. Such synergy promises safe, potent eradication of KRAS-mutant liver tumors [123].

## Important mutations associated with ferroptosis

KRAS, TP53, and BRAF mutations significantly shape ferroptosis regulation across cancers, influencing redox balance, lipid metabolism, and cell survival, thus affecting ferroptosis-based therapy responses. Ferroptosis, driven by iron-mediated lipid peroxidation, was initially tied to RAS-mutant cancers due to elevated oxidative stress and altered lipid profiles. Early studies suggested RAS mutations sensitize cells to ferroptosis, but later findings clarified they alone are insufficient, with tumor microenvironment and metabolic adaptations modulating susceptibility [33]. KRAS-mutated tumors evade ferroptosis via MAPK-driven NRF2 activation, upregulating FSP1 to regenerate ubiquinol, countering lipid peroxidation and promoting survival. Combining ferroptosis inducers with FSP1 inhibitors offers a therapeutic strategy [124]. TP53 mutations variably alter ferroptosis by modulating antioxidant and lipid-handling proteins. In TP53-null H1299 cells with hotspot mutations, deferoxamine upregulated TFRC, but mutants showed fewer proteomic changes. Most mutants slightly increased GPX4 (except R175H), with TXNRD1 highest in R175H. Elevated SCD1 in wild-type and R282W TP53 linked to lipotoxicity resistance, highlighting lipid metabolism’s role [125]. Hotspots like R273H and R175H increase ferroptosis sensitivity, unlike R282W, with inconsistent TFRC/FTH1 changes suggesting complex mechanisms [126]. Radiotherapy activates p53, repressing SLC7A11 to promote ferroptosis; p53-deficient cells resist, but inducers like erastin restore sensitivity, improving radiotherapy outcomes [127]. Acetylation-defective TP53-4KR mutants lack ferroptosis and other functions but delay tumors via mTOR suppression, unlike TP53-5KR, suggesting mTOR inhibitors for TP53-deficient cancers [128]. PTEN regulates ferroptosis via PI3K/AKT. Functional PTEN limits SLC7A11, increasing ferroptosis susceptibility. PTEN loss stabilizes NRF2, enhancing glutathione and resistance [129]. Hyperactive mTORC1 in PTEN-deficient cancers upregulates SCD1, reducing lipid peroxides. Targeting this pathway could enhance ferroptosis therapy [130].

## Conclusion

Ferroptosis, an iron-dependent cell death mechanism driven by lipid peroxidation, is a promising therapeutic strategy for cancer treatment. This review explores mutations in various cancers that modulate ferroptosis susceptibility or resistance, offering insights into their role in cancer progression and therapy resistance. Mutations in EGFR like as exon 19 deletions, L858R, KRAS, and TP53, often increase oxidative stress and lipid peroxidation, enhancing ferroptosis sensitivity. For instance, KRAS mutations in lung and colorectal cancers elevate ROS, making tumors vulnerable to ferroptosis inducers when GPX4 is compromised. Similarly, IDH1 mutations in gliomas promote ferroptosis susceptibility through metabolic reprogramming. Conversely, mutations in KEAP1, NRF2, TP53, and SLC7A11 confer resistance by bolstering antioxidant defenses, such as increased glutathione synthesis or iron sequestration. KEAP1 mutations, for example, activate NRF2, upregulating antioxidant genes to prevent lipid peroxidation, while TP53 mutations stabilize GPX4, enabling tumor survival under oxidative stress.

Despite these insights, ferroptosis-targeting strategies face significant challenges. The effects of mutations are context-dependent; TP53 mutations such as R175H may enhance ferroptosis in some cancers but not others, varying by tumor microenvironment or co-occurring mutations. KRAS-mutant tumors may develop rapid resistance via NRF2-FSP1 activation. Tumor heterogeneity, as seen in EGFR-mutant lung cancers, drives incomplete responses. Current inducers like RSL3 lack specificity, risking toxicity in healthy cells (e.g., neurons). Adaptive resistance, such as alternative antioxidant pathways in SLC7A11-mutant cancers, further limits efficacy. The absence of mutation-specific biomarkers complicates patient stratification, and pre focus hinders understanding of clinical pharmacokinetics and long-term outcomes.

While ferroptosis holds therapeutic potential, addressing these challenges, context-dependent mutation effects, non-specific inducers, adaptive resistance, and biomarker gaps requires further research. Comprehensive clinical trials and mutation-specific strategies are essential to translate ferroptosis-based therapies into effective cancer treatments.

## Data Availability

The original contributions presented in the study are included in the article.
